# Unraveling the role of UilS, a urea-induced acyl-homoserine lactonase that enhances *Serratia marcescens* fitness, interbacterial competition, and urinary tract infection

**DOI:** 10.1128/mbio.02505-24

**Published:** 2024-10-30

**Authors:** Marisel R. Tuttobene, Brayan S. Arango Gil, Gisela Di Venanzio, Javier F. Mariscotti, Rodrigo Sieira, Mario F. Feldman, María Soledad Ramirez, Eleonora García Véscovi

**Affiliations:** 1 Instituto de Biología Molecular y Celular de Rosario, Consejo Nacional de Investigaciones Científicas y Tecnológicas, Universidad Nacional de Rosario, Rosario, Argentina; 2 Department of Molecular Microbiology, Washington University School of Medicine, Saint Louis, Missouri, USA; 3 Fundación Instituto Leloir—IIBBA CONICET, Buenos Aires, Argentina; 4 Department of Biological Science, Center for Applied Biotechnology Studies, College of Natural Sciences and Mathematics, California State University Fullerton, Fullerton, California, USA; Emory University School of Medicine, Atlanta, Georgia, USA

**Keywords:** *Serratia marsescens*, lactonase, quorum quenching, pathogenesis, bacterial virulence

## Abstract

*Serratia marcescens*, a member of the Enterobacteriaceae family, is an opportunistic human pathogen and a frequent cause of urinary tract infections. Clinical isolates often exhibit resistance to multiple antibiotics, posing challenges for successful treatment. Understanding its pathogenic mechanisms is crucial for elucidating new potential targets to develop effective therapeutic interventions and manage *S. marcescens* infections. This work identifies urea-induced lactonase of *Serratia* (UilS), a lactonase encoded in the *S. marcescens* RM66262 strain isolated from a patient with a urinary tract infection. The study explores the bacterium’s response to urea, a major component of urine, and its impact on *uilS* expression. We found that UilS degrades acyl-homoserine lactones (AHL) autoinducers traditionally associated with quorum sensing mechanisms. Surprisingly, UilS is able to degrade self and non-self AHL, exhibiting quorum-quenching activity toward *Pseudomonas aeruginosa*. We found that LuxR regulates *uilS* expression that is enhanced in the presence of AHL. In addition, urea-dependent induction of UilS expression is controlled by the transcriptional response regulator CpxR. UilS confers fitness advantage to *S. marcescens*, especially in the presence of urea, emphasizing the adaptive plasticity of strains to modulate gene expression based on environmental signals and population density. We also discovered a novel bacterial killing capacity of *S. marcescens* that involves UilS, indicating its importance in the interspecies interaction of *Serratia*. Finally, we found that a *uilS* mutant strain displays attenuated colonization in a mouse model of catheter-associated urinary tract infection. *uilS* is present in clinical but absent in environmental isolates, suggesting an evolutionary adaptation to host-specific selective pressures.

**IMPORTANCE:**

This work reveals the acyl-homoserine lactonase urea-induced lactonase of *Serratia* as a novel virulence factor of *Serratia marcescens*, unraveling a potential target to develop antimicrobial strategies and shedding light on the complex regulatory network governing pathogenicity and adaptation to host environments.

## INTRODUCTION

Bacteria are remarkable in their ability to sense the cell density of their proliferating populations and regulate gene expression accordingly. This sensing capacity extends not only to homogeneous bacterial populations but also to mixed populations, where bacteria interact, enabling them to cooperate and share resources, or to compete in a common ecological niche (1). This survival strategy relies on communication mechanisms collectively known as quorum sensing (QS). The simplest QS architecture consists of two key genes: one encoding a synthase enzyme, often of the LuxI-type, responsible for producing an autoinduction molecule, and another encoding a transcriptional response regulator, typically a LuxR-type protein (2). The response regulator is usually activated upon binding to the autoinducer, thereby amplifying the QS response by stimulating the transcription of both *luxR* and *luxI* genes, establishing an autoregulatory amplification cycle. In Gram-negative bacteria, the QS signal molecules are classically acyl-homoserine lactones (AHLs), distinguished by the length and chemical properties of their acyl side chains. AHLs can traverse the bacterial envelope either through diffusion or by active transport, accumulating in the extracellular milieu (3). The concentration of AHL in the extracellular space equilibrates with the intracellular concentration and is therefore a good indicator of a bacterial monoculture population (4).

In addition to regulating QS-encoding genes, the response regulator modulates the expression of other species-specific genes, such as those involved in sporulation, conjugation, virulence, antibiotic production, and biofilm formation. QS signaling can be downregulated when the AHL concentration falls below a defined threshold level due to dilution or disruption of the signaling process (5). The disruption of QS signaling, also known as quorum quenching (QQ), encompasses diverse mechanisms. Such mechanisms include the enzymatic deactivation of AHL molecules by lactonases (that break the lactone ring), acylases (that hydrolyze the acyl chain bound to the lactone ring), reductases (that reduce oxo-chemical groups), or cytochrome oxidases (that oxidize the acyl chain) (5, 6).

*Serratia marcescens*, an opportunistic pathogen within the Enterobacterales, is associated with a wide range of pathologies that depend on the infection’s entry portal and the patient’s immune condition (7). Additionally, clinical isolates of *Serratia* spp. are often multidrug-resistant (8). Therefore, elucidating the underlying mechanisms enabling this pathogen to adapt to the host’s environmental conditions is imperative, along with exploring novel therapeutic strategies for treating *Serratia* infections.

*S. marcescens* strains possess QS systems encoded in their genomes and utilize various homoserine lactones (HSLs), such as C4-HSL, C6-HSL, and C8-HSL, as signal molecules to regulate the production of an extensive array of virulence factors, including lipase, protease, chitinase, nuclease, siderophore, and biofilm formation (9–13). However, up to the moment, QS and QQ regulatory circuits linked to *S. marcescens* virulence in mammalian hosts have been poorly characterized.

*S. marcescens* is frequently isolated from patients with urinary tract infections. Indeed, this pathogen ranks worldwide in the eighth position as an etiological agent of urinary tract infections (14, 15). Taking into account that the main component of normal urine is urea, in an average concentration of 0.33 M (16), we conducted a search for urea-modulated gene expression using the strain *S. marcescens* RM66262 isolated from a patient undergoing urinary tract infection in a public hospital setting (17, 18).

In this study, we identified an acyl-homoserine lactonase in *S. marcescens*, which we termed urea-induced lactonase of *Serratia* (*uilS*), that showed increased expression when grown in the presence of urea. We found that urea and AHL concentration are input signals that induce UilS expression and determined that both CpxR and LuxR transcriptional regulators are required to govern *uilS* expression in response to these stimuli. Furthermore, we found that UilS provides *S. marcescens* with a fitness advantage both *in vitro* and in a murine catheter-associated urinary tract infection (CAUTI) model. Moreover, the phylogenetic analysis of the available whole-genomes of *S. marcescens* strains revealed that *uilS* is conserved in the clinical ones, while *uilS* homologs are absent in most environmental isolates.

## RESULTS

### *uilS* is a lactonase-encoding gene whose expression is modulated by urea

**Fig 1:**
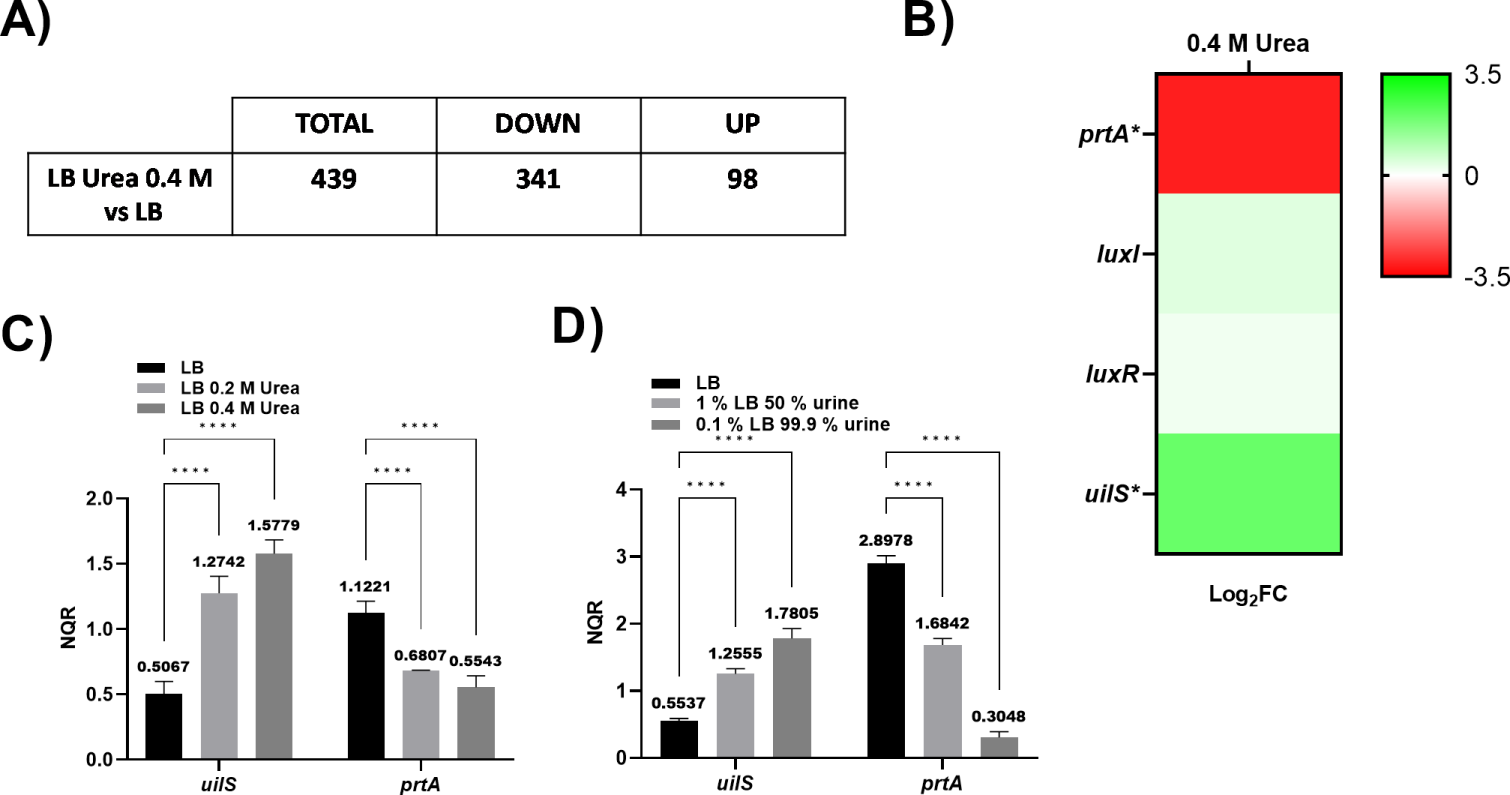
Urea-dependent modulation of *S. marcescens* gene expression. (**A**) Results of RNA-seq indicate DEGs whose expression is modulated by 0.4 M urea. (**B**) The heatmap outlines the differential expression of *prtA*, *luxI*, *luxR*, and *uilS* in the analyses. The scale goes from −3.5 to 3.5. The asterisks represent the DEGs (adjusted *P* < 0.05) with log2fold change >1. (**C and D**) Quantitative reverse transcription-PCR (qRT-PCR) analysis of *uilS* and *prtA* of *S. marcescens* RM66262 cultured in LB broth, LB broth supplemented with 0.2 or 0.4 M urea (**C**) or cultured in LB broth, 1% LB 50% urine, or 0.1% LB 99.9% urine (**D**). The data presented are the mean ± SD of normalized relative quantities (NRQs) derived from transcript levels calculated using the qBASE method. The mean NRQ values are shown above each bar. Three independent samples were used, and two technical replicates were performed for each sample. Statistical significance was determined using a two-way analysis of variance (ANOVA) followed by Tukey’s multiple comparison test. Asterisks indicate the significance levels for the statistical analysis: ∗, *P* < 0.05; ∗∗, *P* < 0.01; ∗∗∗, *P* < 0.001; and ∗∗∗∗, *P* < 0.0001; the analysis was performed using GraphPad Prism (GraphPad Software, San Diego, CA, USA). Data are presented as mean ± SD.

The *S. marcescens* RM66262 strain is non-pigmented and urease negative as its genome lacks the enzymatic machinery responsible for the production of active urease (encoded by the *ureABIEFGH* operon in urease-positive bacteria). Therefore, we hypothesized that urea could be used as environmental signal rather than a metabolic substrate. To identify *S. marcescens* RM66262 genes regulated by urea, we first performed a transcriptomic analysis of bacteria grown with and without urea. For the RNA-seq analysis, we employed 0.4 M urea, which falls within the average concentration found in normal urine (16). An false discovery rate (FDR)-adjusted *P* value of <0.05 and log2-fold change >1 was considered as a differentially expressed gene (DEG). The complete data set revealed 98 upregulated and 341 downregulated DEGs with 0.4 M urea [Fig. 1A and see Table S1 at (https://ibr-conicet.gov.ar/wp-content/uploads/2024/09/mBio-Tuttobene-et-al-Table-S1.xlsx)]. From the upregulated genes, we chose a gene, annotated as a dienelactone hydrolase by the RAST server (19), which we designated *uilS* for urea-induced lactonase of *Serratia*. We prioritized this gene for further investigation as its mRNA expression is induced twofold under 0.4 M urea-Luria-Bertani (LB) growth conditions compared to LB alone (Fig. 1B). No previous dienelactone hydrolase has been characterized in clinical *S. marcescens* isolates, potentially involved in acyl-homoserine lactones hydrolysis and thus in quorum quenching mechanisms, which expression is induced by a condition found in the human urinary tract (the environmental milieu from where the RM66262 was isolated).

To validate the RNA-sequencing results, quantitative reverse transcription-PCR (qRT-PCR) was performed for *uilS* under LB 0.4 M urea, as well as 50% or 99.9% human urine (Fig. 1C and D). As control, the *prtA* gene, whose expression was observed to be strongly repressed by urea, was employed (the *S. marcescens* PrtA serralysin-encoding gene) (20, 21). Because these genes were also significatively affected in response to urea, according to the RNA-seq analysis, the transcriptional expression levels of the genes that encode for the SlpE (22) and SlpD (23) metalloproteases, the regulatory *flhD* gene, the *fliA* gene that encodes for the FliA sigma factor (24), the *fliC* that encodes for the flagellar component FliC, and genes that encode the LipBCD (25), a Type I Secretion System (26) for protein export into the extracellular medium, were evaluated by qRT-PCR. The increase in *uilS* transcriptional levels reached up to 3.2-fold when LB was compared with LB + urine, while *prtA* expression was repressed up to ninefold in LB + urine when compared with LB (Fig. 1D), verifying RNA-seq results. Expression of *fliA*, *fliC*, *flhD*, *splE*, *splD*, *lipB*, and *lipC* genes corroborates the RNA-seq results (see Fig. S1A and B at http://ibr-conicet.gov.ar/wp-content/uploads/2024/09/mBio-Tuttobene-et-al-Supplemental-Material.pdf). Compared with LB, between a 1.5- and sixfold decrease was obtained for the 0.2 M urea condition, while between 4- and 100-fold reductions were observed for 0.4 M urea. Finally, under conditions containing 50% or 99.9% natural urine, between 1.2- and 2.5- (0.2 M urea) or 2- and 25-fold decrease (0.4 M urea) were obtained, respectively, for the previously mentioned genes (see Fig. S1A and B at [http://ibr-conicet.gov.ar/wp-content/uploads/2024/09/mBio-Tuttobene-et-al-Supplemental-Material.pdf]).

**Fig 2:**
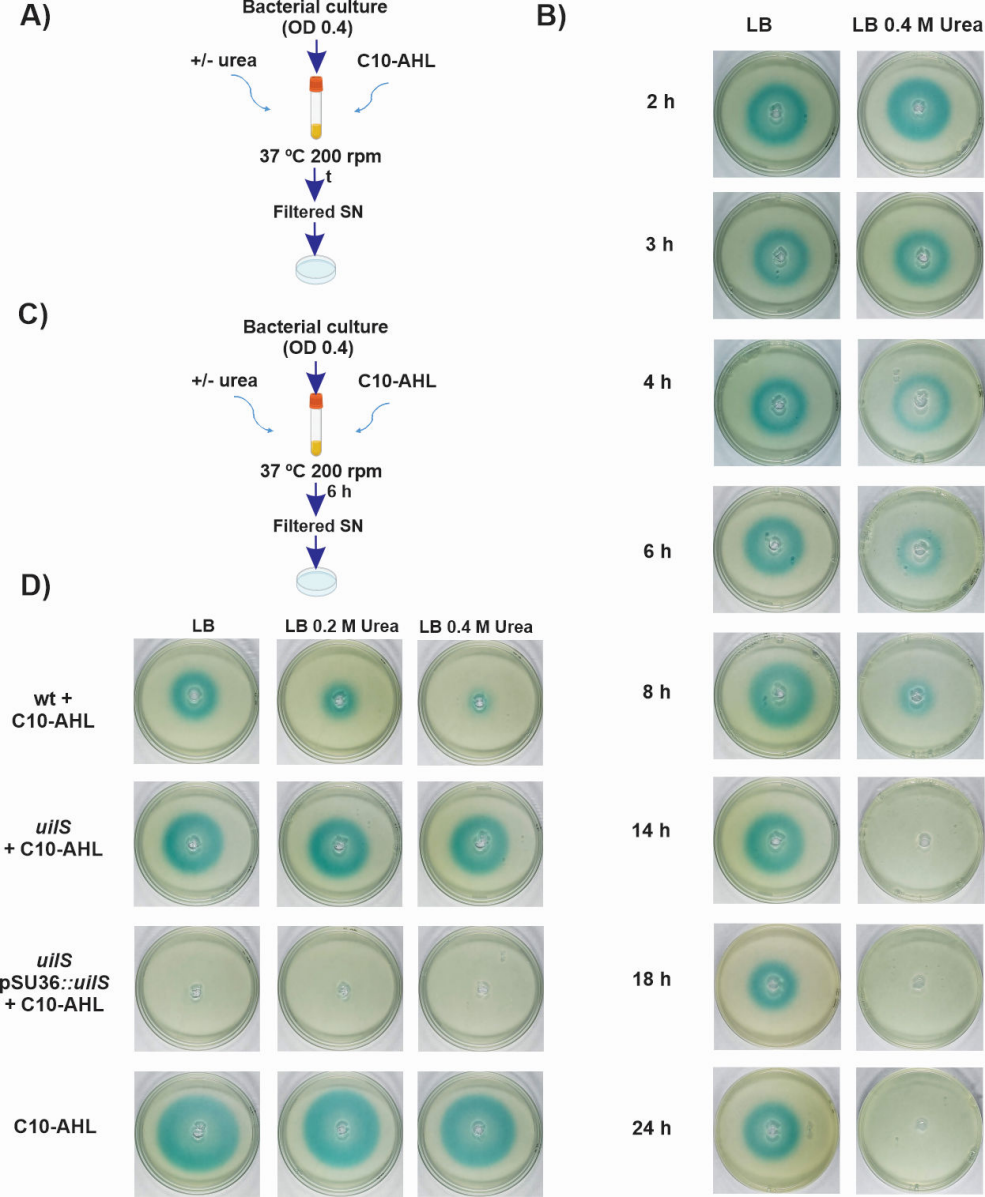
AHLs degradation by urea-induced *uilS* expression. (**A**) Schematic protocol, *S. marcescens* RM66262 strain was inoculated in LB broth supplemented with C-10 AHLs in the presence or absence of 0.4 M urea at 37°C 200 rpm. At the indicated times (see in B), aliquots were taken, and the presence of AHLs was determined in the supernatant (SN) filtered using the *A. tumefaciens* NT1 (pZLR4) biosensor (that produces 5,5′-dibromo-4,4′-dichloro-indigo as a result of the presence of C6–C12 AHLs). (**B**) Results of the experiment described in A, after incubating the plates at 30°C for 24 h. (**C**) Schematic protocol, bacterial cultures were incubated for 6 h shaking in LB or urea-LB medium supplemented with exogenously added C10-AHL followed by the *A. tumefaciens* biosensor plate assay. (**D**) Results of the experiment described in C. AHLs were determined using the biosensor assay in filtered SN of wt, *uilS,* and *uilS* pSU36::*uilS* strains grown in the presence of C-10 AHL and supplemented without or with 0.2 or 0.4 M urea. The standard C-10 AHL was incubated in LB broth under the same conditions as a negative control. Plates were inspected and photographed after 24 h at 30°C. Representative results of three independent experiments are shown.

*uilS* encodes a protein of 31.5 kDa predicted to contain an α-β fold hydrolase domain (Conserved Domain Database, domain architecture ID 1002311). UilS amino acid sequence shares 28% identity with AidA quorum quenching protein, an acyl-homoserine lactonase from *Acinetobacter baumannii* (NCBI, Locus CAI6145363) (27). This prompted us to investigate whether this enzyme could be involved in the degradation of AHLs. For this, we employed the *Agrobacterium tumefaciens* NT1 (pZLR4) biosensor strain, which produces 5,5'-dibromo-4,4'-dichloroindigo (blue color) when co-incubated with bacteria that release AHLs of 6- to 12-carbon length (28). The assay was performed over a 24 h time course, in the presence of a fixed concentration of exogenously added N-Decanoyl-L-homoserine lactone (C10-AHL), with or without the addition of urea to the culture medium. In contrast to the steady color intensity and size of the blue halo observed in LB up to 24 h post-inoculation, in the presence of urea, the blue halo gradually diminished in size over time, disappearing completely after 14 h post-inoculation (Fig. 2A and B). This indicates the presence of a urea-activated, *S. marcescens*-dependent factor that degrades C-10 AHL. It also shows that this factor increases its concentration or activity with the increase in bacterial cell density.

Next, to assess whether UilS was responsible for the observed phenotype, the *A. tumefaciens* biosensor *traG-lacZ* sensor plate-assay (28) was performed with an *S. marcescens* RM66262-derived, *uilS-*deleted mutant strain. Bacteria were incubated for 6 h in LB or urea-LB medium and supplemented with exogenously added C10-AHL, followed by the plate-biosensor assay (Fig. 2C and D). UilS-dependent urea-induced degradation of AHL was lost in the *uilS* mutant (a steady blue halo not affected by the addition of urea, similar to the negative control in which no bacteria was added in the assay), and it was restored in the *uilS* complemented strain regardless of the urea concentration used as it constitutively expresses *uilS* from the pSU36::*uilS* plasmid (Fig. 2D). No difference in growth capacity or rate was detected when comparing the growth curves of the wild type, the *uilS* mutant, and the complemented strains, in LB, LB added with urea or with urine (see Fig. S1C and D at http://ibr-conicet.gov.ar/wp-content/uploads/2024/09/mBio-Tuttobene-et-al-Supplemental-Material.pdf). Moreover, the *Escherichia coli* TOP10 was used to assess the functionality of the UilS protein overexpressed from pSU36::*uilS*. UilS quorum quenching activity was confirmed using the biosensor *A. tumefaciens* since the halo generated by the sole addition of C10-AHL disappeared when the supernatant of the strain *E. coli* TOP10 pSU36::*uilS* was incubated with C10-AHL (see Fig. S2 at http://ibr-conicet.gov.ar/wp-content/uploads/2024/09/mBio-Tuttobene-et-al-Supplemental-Material.pdf).

Endogenous production of AHL by either wild-type or *uilS S. marcescens* cultures grown in LB with or without urea was also evaluated by employing filtered supernatants of the bacterial cultures and either the *A. tumefaciens* biosensor or the *Chromobacterium violaceum* VIR07 biosensor. Strain VIR07 is an AHL-deficient mutant, which does not produce violacein unless long-chain AHLs (C10-C16) are exogenously added but is inhibited by short-chain AHLs [C4–C8; (29)]. Neither the blue halo or violacein was detected when these reporter strains were co-incubated with the *S. marcescens* wild-type strain, while the *uilS* mutant produced strong purple or blue halos, respectively, depending on the biosensor strain (see Fig. S3A through C at http://ibr-conicet.gov.ar/wp-content/uploads/2024/09/mBio-Tuttobene-et-al-Supplemental-Material.pdf). The results indicate that UilS is responsible for the degradation of endogenous long-chain AHLs. It is of note that the lactonase produced by the wild-type strain was able to completely degrade the endogenously produced AHLs, in either the presence or absence of urea.

To further investigate UilS action, bacteria-free culture supernatants obtained either from the *S. marcescens* wild-type, *uilS* mutant, or pSU36::*uilS-*harboring strains were tested. These supernatants were unable to degrade exogenously provided AHL (see Fig. S3D and E at http://ibr-conicet.gov.ar/wp-content/uploads/2024/09/mBio-Tuttobene-et-al-Supplemental-Material.pdf), indicating cytoplasmic localization. This is in agreement with a bioinformatics comparative analysis of bacterial QQ AHL acylases and lactonases, indicating that, while acylases are predominantly periplasmic, lactonases are predicted to be localized to the bacterial cytoplasm (30).

**Fig 3:**
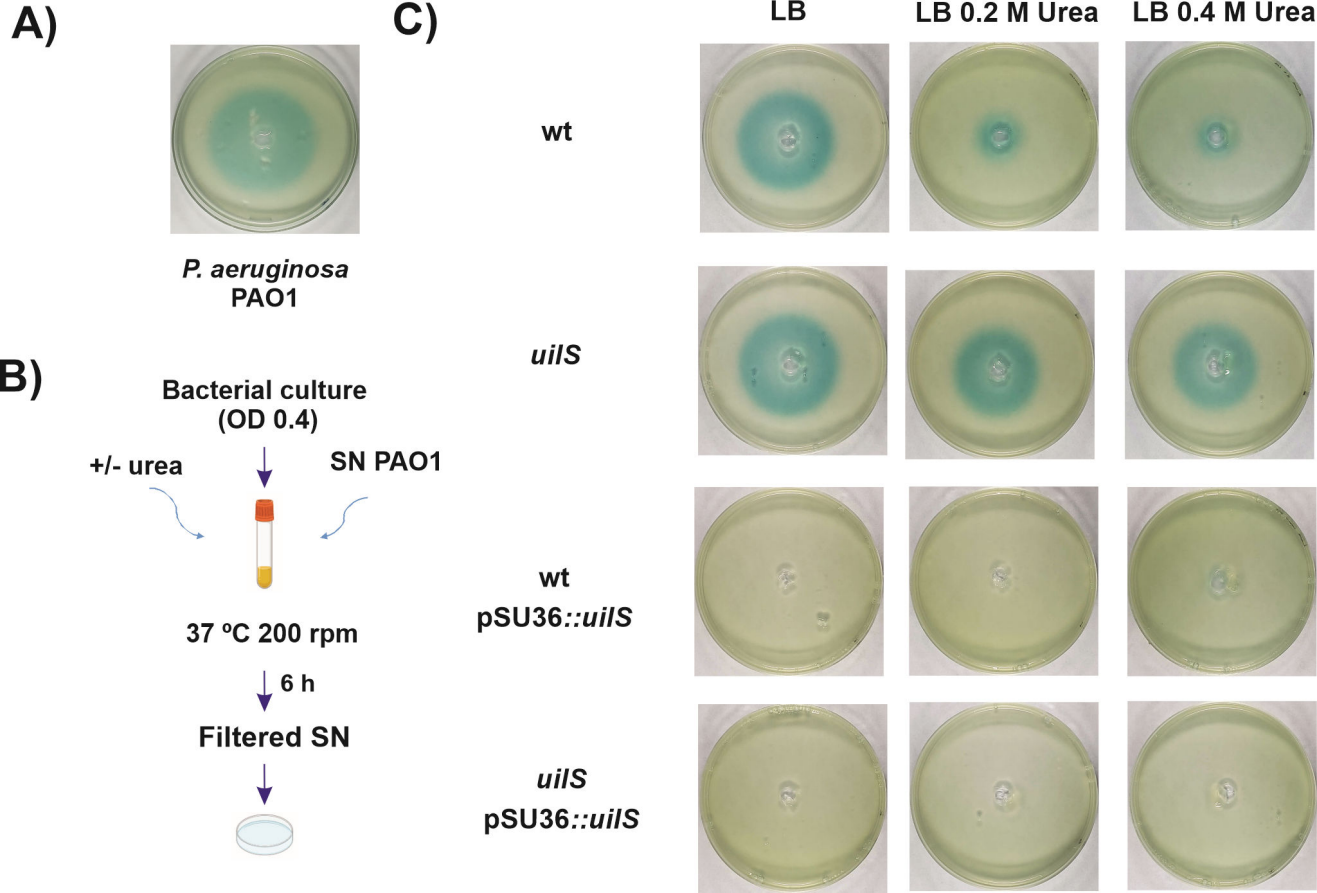
Urea-induced *uilS* degradation of AHL produced by *P. aeruginosa*. (**A**) AHLs produced by *P. aeruginosa* PAO1 strain determined by *A. tumefaciens* biosensor assay. (**B**) Scheme of the protocol, *S. marcescens* strains were incubated for 6 h shaking in LB or urea-LB medium supplemented with the filtered SN of PAO1 strain added followed by the *A. tumefaciens* biosensor assay. (**C**) AHLs were determined using the biosensor assay using filtered SN of PAO1 preincubated with bacteria culture of wt, *uilS*, wt pSU36*::uilS*, and *uilS* pSU36::*uilS* strains grown supplemented without or with 0.2 or 0.4 M urea. Plates were inspected and photographed after 24 h at 30°C. Representative results of three independent experiments are shown.

We also found that *Serratia* urea-induced *uilS* was able to degrade AHL produced by the *Pseudomonas aeruginosa* PAO1 strain (that produces N-(3-oxododecanoyl) homoserine lactone (3–O–C12–AHL) and N-butyryl homoserine lactone (C4–AHL) (31) (Fig. 3A through C). This degradation followed the same kinetics as the one observed for the degradation of exogenous AHL by *S. marcescens* (compare Fig. S4A and B at http://ibr-conicet.gov.ar/wp-content/uploads/2024/09/mBio-Tuttobene-et-al-Supplemental-Material.pdf; with Fig. 2). On the other hand, the culture spent media from the wild-type, *uilS*, wild-type/pSU36::*uilS*, or *uilS*/pSU36::*uilS S. marcescens* strains were unable to degrade the AHL produced by *P. aeruginosa*, even when urea was added to the culture. This strengthened the notion that the UilS lactonase is not secreted (see Fig. S4C and D, at http://ibr-conicet.gov.ar/wp-content/uploads/2024/09/mBio-Tuttobene-et-al-Supplemental-Material.pdf), and no other secreted exoenzyme from *Serratia* is able to degrade these AHLs.

### *S. marcescens uilS* is a member of the LuxR regulon

**Fig 4:**
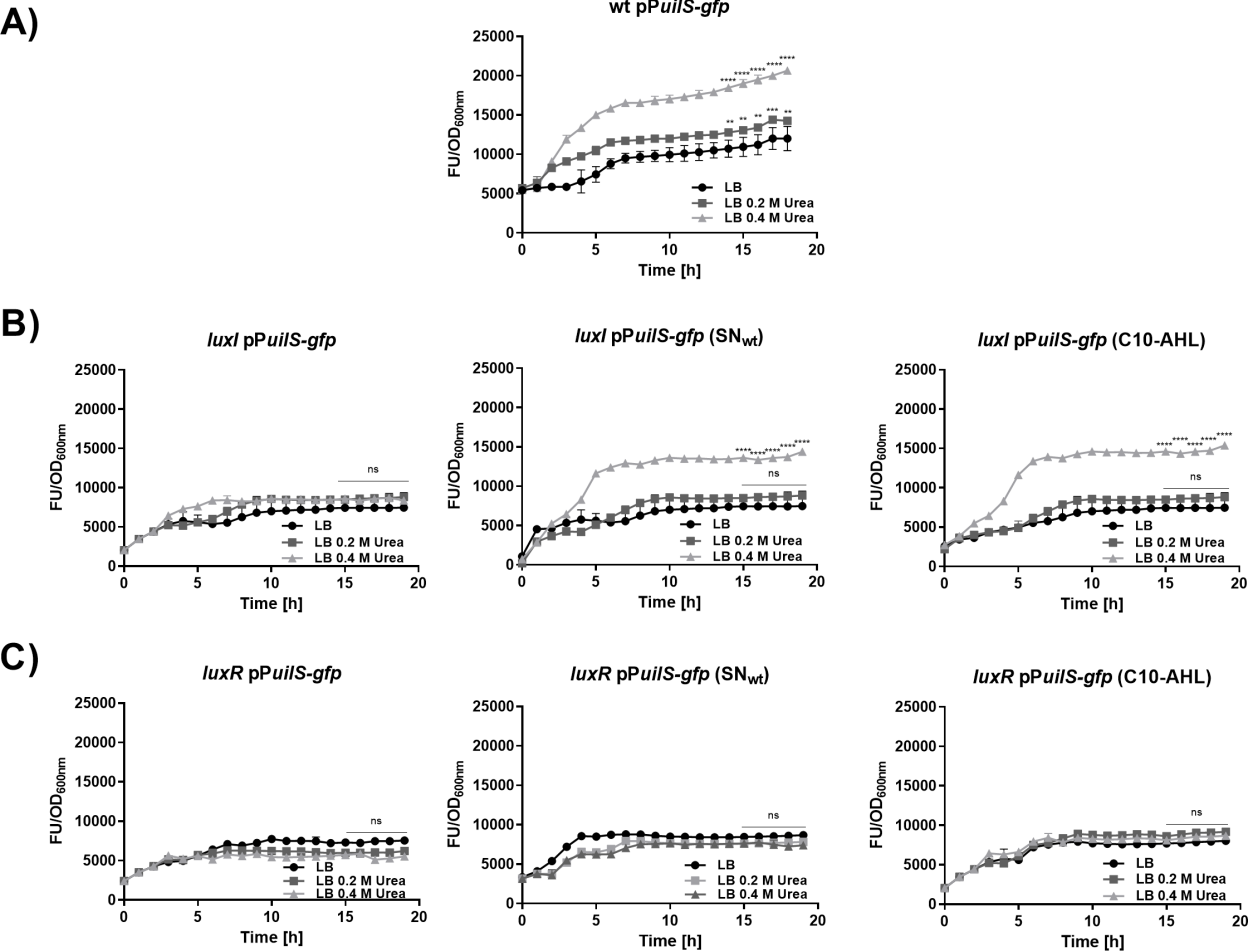
Transcriptional expression of *uilS*. Bacteria were grown for 18 h in LB or LB supplemented without or with 0.2 or 0.4 M urea, in 96-well microplates, at 37°C with agitation. Transcriptional activity was calculated as the ratio of green fluorescent protein (GFP) fluorescence values and OD600 measured from the *S. marcescens* wild-type (wt; FU/OD600) (**A**) *luxI* strain supplemented with wt strain or with C-10 AHL, (**B**) *luxR* strain supplemented with filtered SN from the wt or with C10-AHL, and (**C**) carrying the P*uilS*-gfp reporter plasmids. Means ± SDs from three independent experiments performed in duplicate in each case are shown. Statistical significance (*P* < 0.05) was determined by two-way ANOVA followed by Tukey’s multiple comparison test, comparing each mean (every measured time) with the control LB condition. The last five points are shown. Significance was indicated by *P < 0.05, **P < 0.01, ***P < 0.001, and ****P < 0.0001 employing GraphPad Prism (GraphPad Software, San Diego, CA, USA).

**Fig 5:**
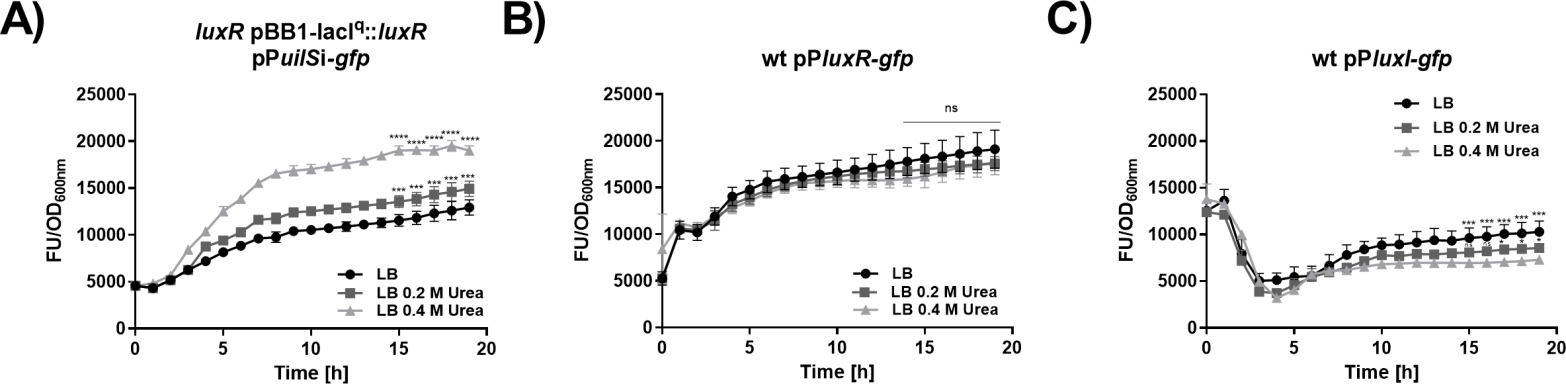
Transcriptional expression of *uilS*, *luxR*, and *luxI*. Bacteria were grown for 18 h in LB or LB supplemented without or with 0.2 or 0.4 M urea, in 96-well microplates, at 37°C with agitation. Transcriptional activity was calculated as the ratio of GFP fluorescence values and OD600 measured from the *S. marcescens luxR* pBB1-*lacI*^q^::*luxR* (FU/OD600) carrying the P*uilS*-gfp reporter plasmids. (**A**) Wild-type *S. marcescens* carrying either the P*luxR*-gfp (**B**) or the P*luxI*-gfp reporter plasmids (**C**). Means ± SDs from three independent experiments performed in duplicate in each case are shown. Statistical significance (*P* < 0.05) was determined by two-way ANOVA followed by Tukey’s multiple comparison test, comparing each mean (every measured time) with the control LB condition. The last five points are shown. Significance was indicated by *P < 0.05, **P < 0.01, ***P < 0.001, and ****P < 0.0001 employing GraphPad Prism (GraphPad Software, San Diego, CA, USA).

To investigate whether *uilS* forms part of the AHL-dependent quorum sensing system of *S. marcescens*, we constructed *uilS-*, *luxI-*, and *luxR-*deleted strains. Transcriptional levels of *uilS* expression were examined by the use of a reporter plasmid that contains the *gfp* gene (that encodes green fluorescent protein) under the control of the *uilS* promoter region (533 bp upstream of the *uilS* ATG translational start codon). The urea-dependent induction profile of *uilS* expression (Fig. 4A) corroborated the results shown in Fig. 2A, indicating an increasing expression level along the time-course of bacterial growth. When the assay was performed in the *luxI* background, urea induction was lost unless either a supernatant from the wild-type strain culture or pure C10-AHL was exogenously added to the culture medium (Fig. 4B). This result suggested that the role of *luxI* in this regulatory mechanism is restricted to the synthesis and provision of AHL. In contrast, although a basal *uilS* expression level is observed in the absence of *luxR*, the inability of the *luxR* strain to induce *uilS* expression could not be compensated neither by the wild-type strain spent supernatant nor by exogenously added C10-AHL (Fig. 4C). *luxR* expression from the inducible pBB1-*lacI*^q^::*luxR* plasmid complemented the *luxR* strain phenotype for the capacity to activate *uilS* transcription in an urea concentration-dependent fashion (Fig. 5A). This indicates that *uilS* expression levels are modulated by an AHL-activated LuxR regulator. However, neither *luxR* nor *luxI* transcriptional expression was affected by the presence of urea in the growth medium (a result also observed in the RNA-seq analysis, see Table S1 at https://ibr-conicet.gov.ar/wp-content/uploads/2024/09/mBio-Tuttobene-et-al-Table-S1.xlsx, and Fig. 1) as verified by using *gfp*-based reporters for both *luxR* and *luxI* genes (Fig. 5B and C).

### CpxR induces *uilS* expression in response to urea

**Fig 6:**
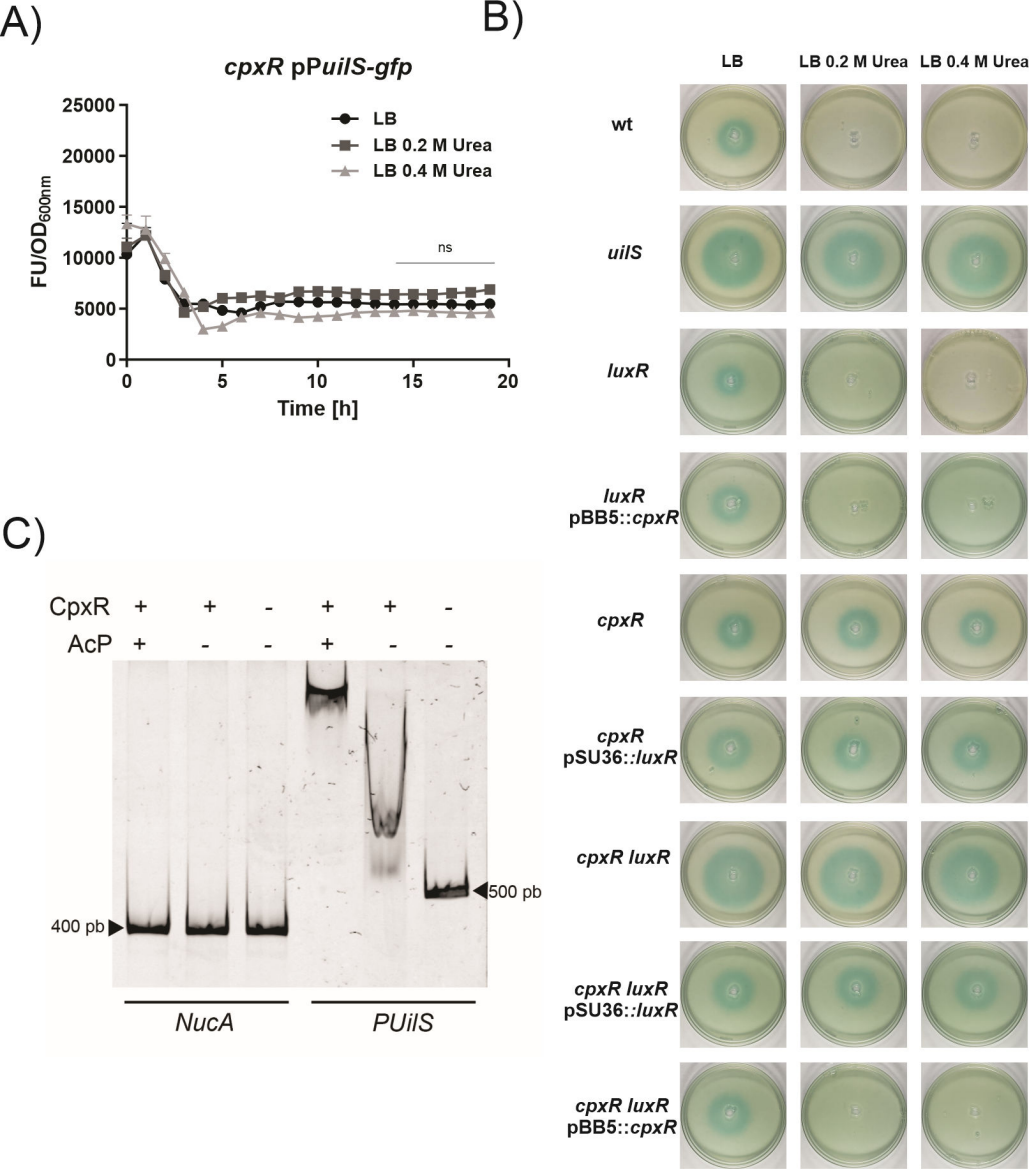
CpxR-dependent urea-modulated induction of *uilS* expression. (**A**) Transcriptional expression of *uilS* in the *cpxR* mutant strain. Bacteria were grown for 18 h in LB or LB supplemented with 0.2 or 0.4 M urea, in 96-well microplates, at 37°C with agitation. Transcriptional activity was calculated as the ratio of GFP fluorescence values and OD600 (FU/OD600) measured from the *cpxR*/P*uilS*-gfp strain. Means ± SDs from three independent experiments performed in duplicate in each case are shown. Statistical significance (*P* < 0.05) was determined by two-way ANOVA followed by Tukey’s multiple comparison test, comparing each mean (every measured time) with the control LB condition. The last five points are shown. Significance was indicated by *P < 0.05, **P < 0.01, ***P < 0.001, and ****P < 0.0001 employing GraphPad Prism (GraphPad software, San Diego, CA, USA). (**B**) Degradation of the AHLs of *P. aeruginosa* PAO1 was determined using the biosensor assay using filtered SN from this strain incubated with wt, *uilS*, *luxR*, *luxR* pBB5::*cpxR*, *cpxR, cpxR* pSU36:*luxR*, *cpxR luxR*, *cpxR luxR* pSU36::*luxR*, and *cpxR luxR* pBB5::*cpxR Serratia* strains grown in LB or LB supplemented with 0.2 or 0.4 M urea (see the experiment scheme in the Fig. 3B). Plates were inspected and photographed after 24 h at 30°C. Representative results of three independent experiments are shown. (**C**) Electrophoretic mobility shift assay (EMSA). Interaction between the DNA promoter region of *uilS* and the CpxR protein is shown; increasing affinity is observed when acetyl phosphate (AcP) is added. No shift was detected when the non-specific probe (NucA) was used.

Because neither LuxR nor LuxI transcriptional levels were altered by the presence of urea in the growth medium, we investigated whether transcriptional response regulators responsive to bacterial stress conditions, belonging to the EnvZ/OmpR, PhoQ/PhoP, Rcs RssA/RssB, or CpxAR two-component systems (32–36), might be involved in the urea-modulated *uilS* expression. While *uilS* expression was not affected by *ompR*, *phoP*, *rcsB*, or *rssB* genetic backgrounds (see Fig. S5 at http://ibr-conicet.gov.ar/wp-content/uploads/2024/09/mBio-Tuttobene-et-al-Supplemental-Material.pdf), the urea-dependent phenotype was abrogated in the *cpxR* mutant strain (Fig. 6A).

This last result was also examined by monitoring the degradation of exogenously added AHL produced by *P. aeruginosa* PAO1 strain by *S. marcescens* wild-type and mutant strains with the *A. tumefaciens traG-lacZ* biosensor assay. In the wild type, the *luxR*, and the *luxR/*pBB5::*cpxR* strains, the AHL halo decreased with the addition of urea to the growth medium, indicating that LuxR is not the regulator that controls *uilS* expression in response to urea (Fig. 6B). In contrast, the *cpxR* and *cpxR*/pSU36::*luxR* strains could not recover the wild-type urea-mediated induction of AHL degradation. While the *cpxR luxR* double mutant strain lost the capacity to degrade AHL and equaled the *uilS* strain inability to degrade AHL, *in trans* expression of *cpxR* from the pBB5::*cpxR* plasmid (but not the *luxR* expression from pSU36::*luxR*) recovered the urea-mediated induction of AHL degradation (Fig. 6B). In sum, our results demonstrate that there is a basal *uilS* expression level in the wild-type strain even in the absence of *luxR,* that LuxR stimulates *uilS* transcription in the presence of AHL, and that CpxR is the response regulator that mediates the urea-dependent modulation of *uilS* expression.

To explore whether CpxR interacts directly with the putative regulatory region of *uilS*, a DNA electrophoretic mobility shift assay (EMSA) was performed. A DNA oligonucleotide that comprises 500 bp upstream the translational ATG of *uilS* and an affinity purified His-tagged CpxR protein was co-incubated and resolved by PAGE. CpxR provoked a shift in the mobility of the *uilS* promoter oligonucleotide, while no shift was observed when a non-specific *nucA* oligonucleotide was used (Fig. 6C). The addition of the CpxR phosphate donor acetyl-phosphate further enhanced the mobility shift, suggesting that, as previously described (37), the phosphorylated/activated form of CpxR would be able to dimerize, and as a consequence, an enhanced affinity of CpxR for its target DNA would be achieved. This result demonstrates that CpxR is able to directly recognize and specifically interact with the promoter region of *uilS*.

### UilS endows *S. marcescens* with fitness advantage and killing capacity

**Fig 7:**
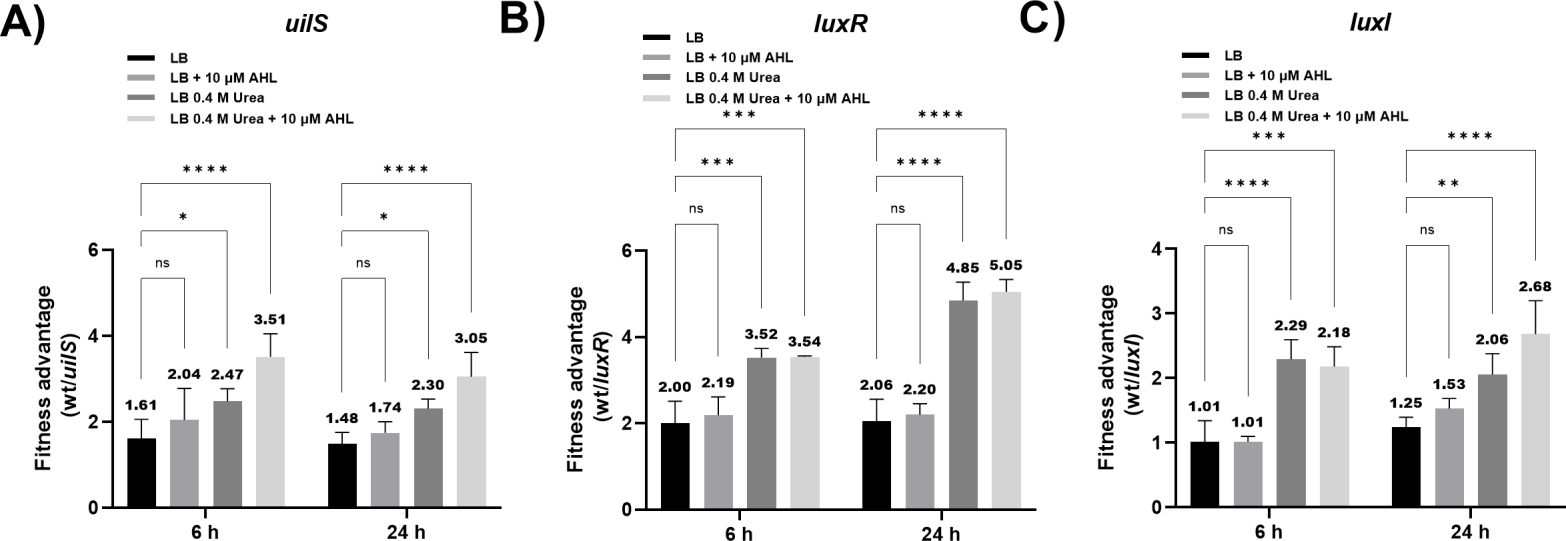
Fitness advantage conferred by UilS expression. Recovery of viable *S. marcescens* cells after 6 h or 24 h of co-culture between wild-type (wt) and *uilS* (**A**), wt and *luxR* (**B**), and wt and *luxI* (**C**) at 37°C in LB, LB 0.4 M urea, LB 10 µM C10-AHL, or LB 0.4 M urea 10 µM C10-AHL, with an initial ratio of 1:1. The fitness advantage was calculated as the ratio as wild-type-to-mutant (CFU wt/CFU mutant) divided by the corresponding ratio in the inoculum. Results from three independent experiments are shown. The mean values are shown above each bar. Statistical significance (*P* < 0.05) was determined by two-way ANOVA followed by Tukey’s multiple comparison test, comparing each mean (every measured time) with the control LB condition. The last five points are shown. Significance was indicated by *P < 0.05, **P < 0.01, ***P < 0.001, and ****P < 0.0001 employing GraphPad Prism (GraphPad Software, San Diego, CA, USA).

To examine whether UilS expression could confer fitness advantage to *S. marcescens*, the wild-type strain was co-incubated in a 1:1 relationship with either the *uilS*, the *luxI*, or the *luxR* strains in LB, LB + AHL, LB + urea, or LB + urea + AHL and monitored at 6 h and at 24 h. Each strain was then selected by its distinctive antibiotic resistance, and colony-forming units (CFUs) were quantified. The wild-type strain showed fitness advantage over the *uilS* strain under all the conditions tested (Fig. 7A). The addition of AHL, urea, or urea + AHL increased the augmented CFUs recovered in favor of the wild-type strain, which is consistent with the conditions that stimulate *uilS* expression (Fig. 7A). The *luxR* strain showed similar fitness disadvantages as the *uilS* strain, except that, as can be expected, the addition of AHL did not alter the values observed in LB or LB + urea (Fig. 7B). The *luxI* strain shows fitness disadvantage with respect to the wild type when AHL, urea, or the combination of the two are present in the culture medium, and the difference is more marked when measured at 24 h (Fig. 7C). These last results reinforce our previous observations, indicating that LuxI-dependent production of the AHL signal induces *uilS* expression in a LuxR-dependent manner. Overall, our observations indicate that the conditions that were previously shown to enhance *uilS* expression augment the fitness of the wild-type strain over the strains that cannot fully express UilS.

**Fig 8:**
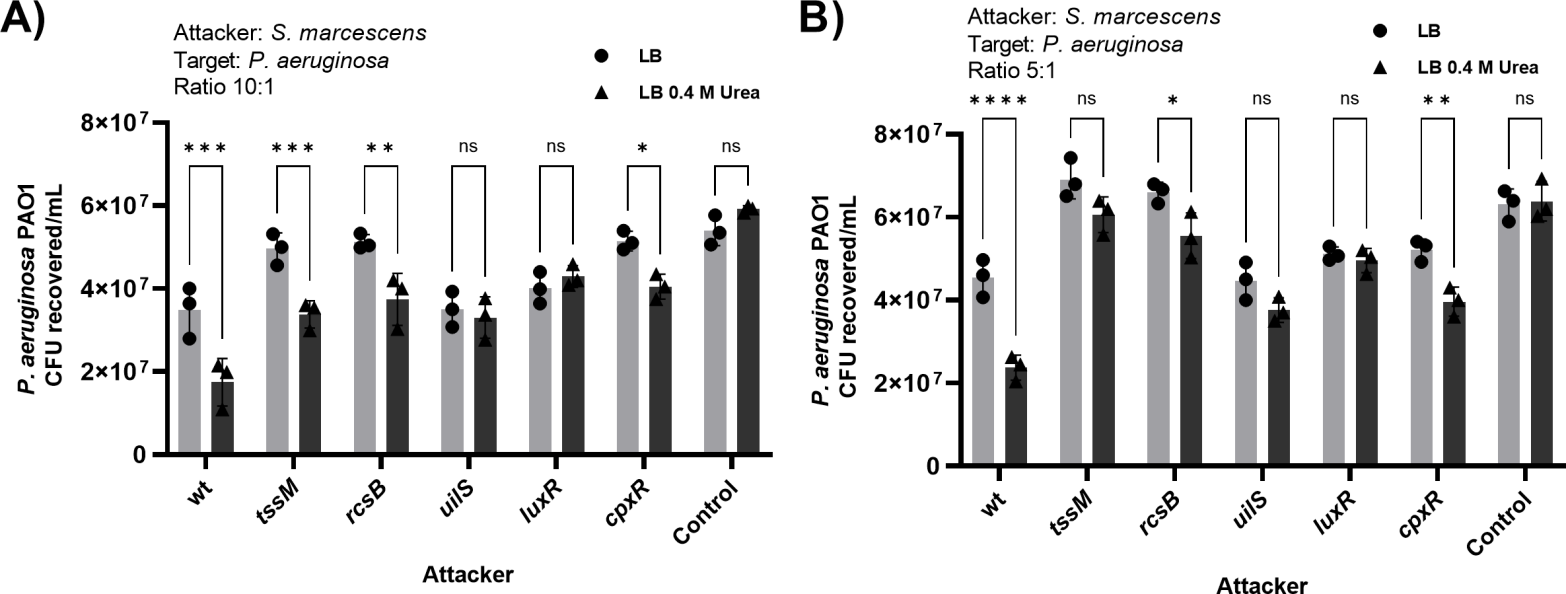
Interspecies killing. Recovery of viable *P. aeruginosa* PAO1 cells (prey) after 6 h of coculture with the indicated *Serratia* strain (attacker) at 37°C in LB or LB supplemented with 0.4 M urea, with an initial ratio of 10:1 (**A**) or 5:1 (**B**) (attacker/pray). *P. aeruginosa* PAO1 mono-culture was used for controls. Average values ± SD from three independent experiments are shown. Statistical significance was determined using a two-way ANOVA followed by Tukey’s multiple comparison test. Asterisks indicate the significance levels for the statistical analysis: *P* < 0.05; ∗∗, *P* < 0.01; ∗∗∗, *P* < 0.001; and ∗∗∗∗, *P* < 0.0001; the analysis was performed using GraphPad Prism (GraphPad Software, San Diego, CA, USA). A *P* < 0.05 was considered significant.

Because the wild-type strain showed adaptive advantages over the *uilS* strain, we also investigated whether *uilS* expression could be beneficial for *S. marcescens* when antagonizing with other bacterial species. To this end, the wild-type and its derived-mutant strains (attackers) were challenged in a killing assay with the *P. aeruginosa* PAO1 strain (pray), using a 5:1 or a 10:1 attacker-to-pray ratio, in the presence or absence of urea. Wild-type *Serratia* was able to lower *P. aeruginosa* CFUs by 1.8-fold (Fig. 8), and this killing capacity was enhanced 3.7-times in the presence of urea. The Type 6 Secretion System (T6SS) has the capacity to secrete and inject lethal effectors to competitor strains, and it is, up to now, the major killing strategy described for *S. marcescens* (38, 39). Neither the *tssM* (impaired in the expression of the T6SS) (39–41) nor the *rcsB* strain (which we previously demonstrated cannot induce T6SS expression in response to the challenge with competing bacteria) (39) lost the urea-dependent increase in their capacity to kill the prey, indicating a T6SS-independent mechanism involved. The expression level of the Hcp protein in the bacterial culture supernatant reflects the active assembly of the T6SS; as analyzed by immunodetection, the Hcp protein levels were not affected by the addition of urea or by the inactivation of *uilS*, *luxI*, or *luxR* (see Fig. S6 at http://ibr-conicet.gov.ar/wp-content/uploads/2024/09/mBio-Tuttobene-et-al-Supplemental-Material.pdf). These results indicated that urea has no influence neither on the T6SS expression or assembly nor on the T6SS-dependent killing ability of *S. marcescens*. The killing capacity of the *uilS or luxR* strains was not altered by the addition of urea to LB, while a minor effect was observed for the *cpxR* mutant (Fig. 8). Because the CpxAR two-component system has been described to regulate a vast array of genes in Enterobacterales (42), our results indicate that urea would be a signal detected by CpxAR in *S. marcescens*. CpxR-dependent expression of not yet characterized genes in addition to *uilS* might also contribute to the killing capacity of *Serratia* (Fig. 8). We also verified that *P. aeruginosa* PAO1 viability was not altered by the addition of urea to the LB medium (Fig. 8A and B, control). In sum, these results indicate that the expression of the urea-induced lactonase UilS contributes not only to *S. marcescens* fitness but also improves *Serratia’s* capacity to successfully cope with an interspecies competition challenge.

### UilS contributes to the pathogenic traits of *S. marcescens*

**Fig 9:**
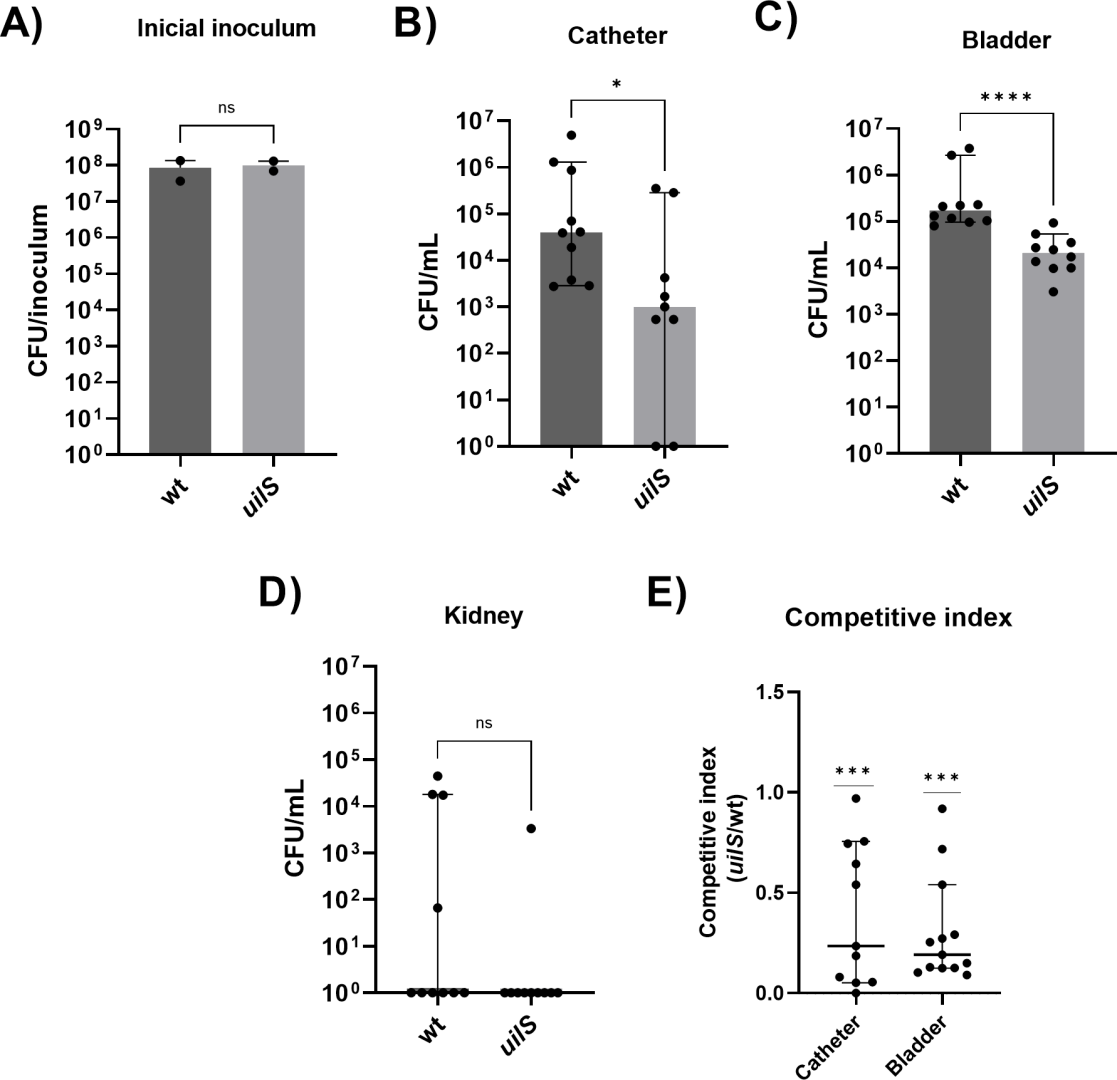
*uilS* role in a mice CAUTI model of infection. Catheter-implanted mice were infected with ~1 × 10^8^ CFU of *S. marcescens* wild-type (wt) and *uilS* strains. (**A**) CFU of initial inoculum. Following 24 h of infection, total number of CFU recovered was determined for catheters (**B**), bladders (**C**), and kidneys (**D**). In B and C, CFU was counted per organ. Each symbol represents an individual mouse. The median with 95 confidence interval is shown. Statistical analyses were performed using the Mann–Whitney *U* test performed using GraphPad Prism (GraphPad Software, San Diego, CA, USA). A *P* < 0.05 was considered significant. (**E**) CI between *uilS* and wild type. CI was defined as the mutant-to-wild-type ratio (CFU mutant/CFU wild type) within the output sample, divided by the corresponding ratio in the inoculum. Statistical significance was evaluated using the one-sample Wilcoxon test using Graph Pad Prism. Asterisks indicate the significance levels for the statistical analysis: *P* < 0.05; ∗∗, *P* < 0.01; ∗∗∗, *P* < 0.001; and ∗∗∗∗, *P* < 0.0001.

In light of the *in vitro* observed benefits conferred by UilS to *S. marcescens*, and considering that the *S. marcescens* RM66262 strain was originally isolated from a patient with a urinary tract infection (17), we investigated the role of *uilS* in a murine model of CAUTI (43). To induce CAUTI, mice underwent transurethral catheterization with a sterile silicone tubing and were immediately inoculated with 1 × 10^8^ CFUs of either the wild-type or *uilS* mutant strains. After 24 h of infection, mice were euthanized, and catheters, bladders, and kidneys were aseptically excised for CFU quantification (Fig. 9A). The mutant was attenuated 40 and 8.33 times in the catheter and bladder, respectively, indicating that *uilS* expression plays a relevant role in the urinary tract infection of *Serratia*, affecting both catheter and bladder colonization, but not in the spread to the kidneys. To further analyze this UilS-dependent phenotype, a competitive index (CI) was carried out by using the CAUTI model of infection and an inoculum of a 1:1 ratio of wild-type and *uilS* strains. Either in catheter or in bladder, the competitive index value obtained was below 0.5 (0.23 for catheter and 0.19 for bladder; Fig. 9E), reinforcing the conclusion that *uilS* is involved in the pathogenicity of *S. marcescens* when colonizing the urinary tract.

### *uilS* homologs are present in *S. marcescens* clinical strains

The phylogenetic analysis based on the DNA sequences of 21 *S*. *marcescens* strains from clinical and environmental sources whose whole-genome sequences are deposited in the NCBI Genome website (https://www.ncbi.nlm.nih.gov/genome) shows that UilS orthologs (amino acid sequences deduced from nucleotide sequences) are present in all clinical strains analyzed and show between 97% and 100% identity with the UilS present in the RM66262 strain genome.

**Fig 10:**
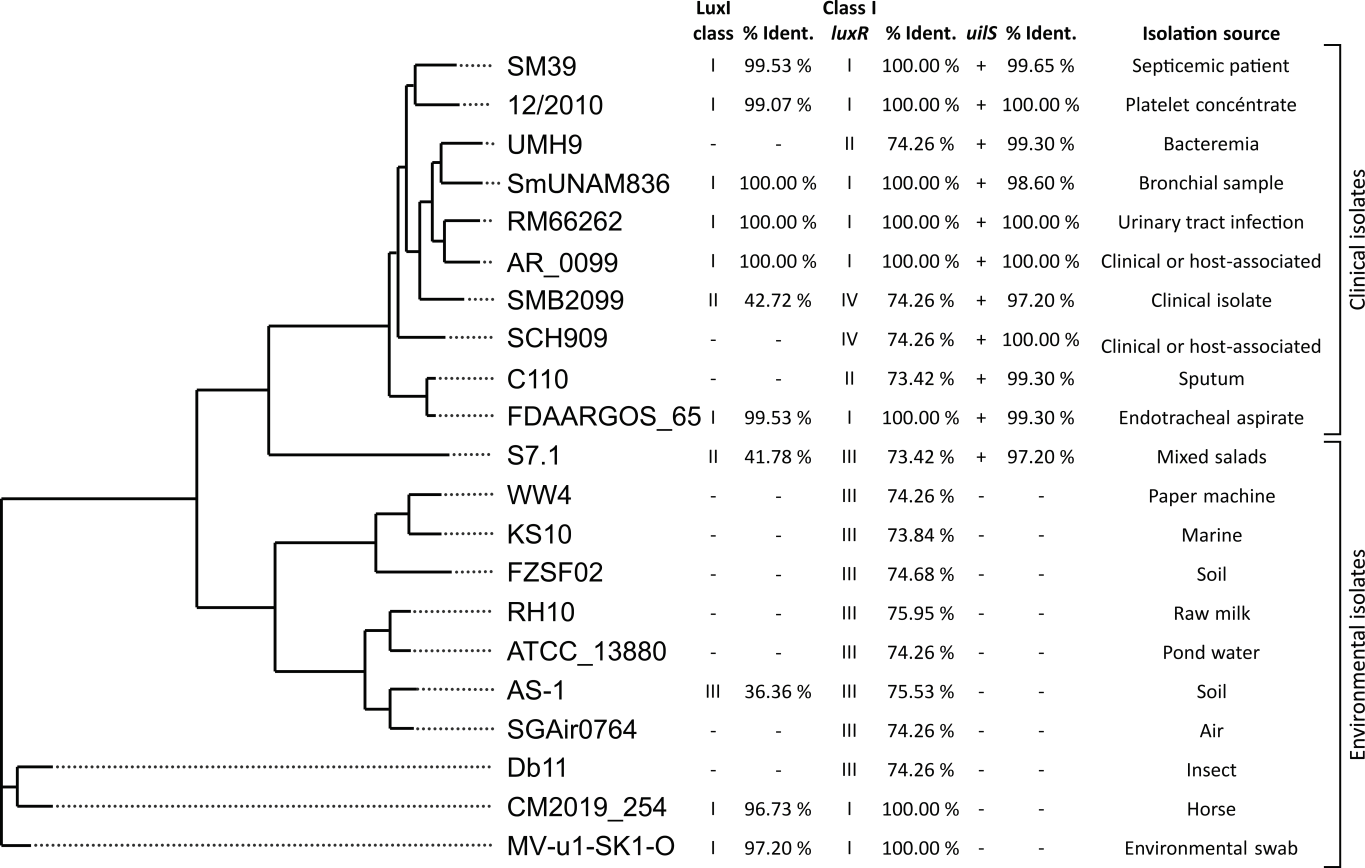
Phylogenetic tree based on the complete genomic sequences of 21 strains of *S. marcescens*. The phylogenetic tree was constructed with the aid of REALPHY 1.13. The classification of *luxI*, gene arrangement around the class I *luxR* homolog, presence of *uilS* gene, percentage of amino acid identity (% Ident.) with strain RM66262, and the isolation source of strains is described on the right side of the tree. Based on the deduced amino acid sequences of the *luxI* genes, LuxI homologs were divided into three classes according to an identity level of 95%, as previously described (37). Class I contains 8 LuxI homologs, Class II contains 2 LuxI homologs, and Class III presents only one LuxI homolog in the AS-1 genome. GenBank database accession numbers for *S. marcescens* strains genomes: AP013063 (SM39), CP053925 (12/2010), CP018923 (UMH9), CP012685 (SmUNAM836), NZ_JWLO00000000 (RM66262), CP027539 (AR_0099), HG738868 (SMB2099), CP063238 (SCH909), CP047691 (C110), NZ_CP026050 (FDAARGOS_65), CP053572 (S7.1), CP003959 (WW4), CP027798 (KS10), CP053286 (FZSF02), CP092461 (RH10), CP072199 (ATCC 13880), AP019009 (AS-1), CP027300 (SGAir0764), HG326223 (Db11), NZ_CP091120 (CM2019_254), and CP085860 (MV-u1-SK1-O).

Strikingly, *uilS* was absent in all but one of the environmental strains examined (Fig. 10). While only some strains harbor a *luxI* gene with no apparent occurrence pattern with respect to the strain source, all of them contain a *luxR* ortholog. The observed synteny of *uilS* and *luxR* aids in the requirement of the LuxR response regulator to allow low levels of *uilS* expression and respond to AHL stimuli, while *luxI*, and thus endogenously produced AHL, would be dispensable for the regulatory mechanism to take place. This observation together with our previously shown results leads us to postulate that *uilS* has been evolutionarily selected to favor the adaptation of the pathogen to conditions found in the host.

The phylogenetic analysis also shows the classification of LuxI and LuxR homologs also searched in the 21 *S*. *marcescens* strains analyzed. The results of the BLAST search revealed that 11 of these 21 genomes contained a *luxI* homolog (Fig. 10).

## DISCUSSION

*S. marcescens* RM66262 was isolated from a patient undergoing a urinary tract infection. To successfully colonize the urinary tract, bacteria must overcome harsh environmental conditions such as the presence of urine, which contains urea in an average concentration of 0.3 M. In this work, we found that *uilS*, encoding for a cytoplasmic lactonase able to degrade AHL autoinducer molecules, is one of the most upregulated genes when *S. marcescens* is exposed to 0.4 M urea.

QQ molecules, including the AHL lactonases, have been almost exclusively associated with the regulation of self or non-self autoinducer’s concentration, and as such, they have been considered a component of QS mechanisms. We determined that UilS possesses AHL degrading activity in the bacterial cytoplasm. Although UilS has the capacity to degrade the AHL autoinducers produced by its own species, it is also able to hydrolyze the AHL released to the extracellular medium by a non-related specie, i.e., *P. aeruginosa*. Therefore, external AHLs should be imported to the bacterial cytosol to be hydrolyzed by UilS. Although there is a basal LuxR-independent expression of *uilS* when bacteria are grown in LB, the presence of AHL enhances *uilS* transcriptional expression in a LuxR-dependent manner. In this context, we also show that *luxI* is dispensable for the LuxR-dependent induction of *uilS* expression when the AHL signal is provided from an exogenous source.

As expected for a canonical LuxR-regulated gene, expression of *S. marcescens* AHL-degrading activity augmented with the increase in bacterial density. However, we found that LuxR was not responsible for the urea-dependent increase of *uilS* transcriptional levels. This became apparent as the *luxR* mutant strain, under urea stimulus, could be induced to degrade the remnant exogenous AHL (Fig. 6B). In accordance with these results, we determined that neither *luxR* nor *luxI* transcriptional levels were altered by the presence of urea in the bacterial growth medium (Fig. 5).

Urea is a well-known chaotropic non-osmotic stressor. To assess the regulatory mechanism involved in the urea-dependent response, we assayed the urea-dependent activation of *uilS* transcription in *ompR*, *phoP*, *rcsB*, *rssB*, or *cpxR* mutant strains backgrounds. These genes encode five crucial transcriptional regulators within the two-component family of signal transduction systems, governing adaptive responses to environmental stresses in gram-negative bacteria. We found that only the inactivation of *cpxR* abolished the urea-dependent induction of *uilS* expression. Cross-complementation assays by *in trans* expression of LuxR or of CpxR in either the *luxR*, *cpxR*, or the *luxR cpxR* mutant strains confirmed that CpxR is the regulator required for the urea-dependent induction of UilS expression. By electrochemical mobility shift assays, we determined that CpxR is able to specifically recognize and interact with the putative regulatory region upstream of *uilS*, and this interaction was enhanced by the addition of the acetyl-phosphate phosphoryl donor, which is known to favor the phosphorylated, activated status of CpxR (21). This result confirmed the direct involvement of CpxR as a key component in the regulatory cascade that governs *uilS* transcriptional regulation. In sum, our results demonstrate that LuxR upregulates a basal-level expression of *uilS* in response to either endogenous or exogenous AHL concentrations, while CpxR operates by further enhancing *uilS* transcriptional levels in response to the concentration of urea in the bacterial environment.

Because UilS expression is able to degrade exogenous AHL in response to autoinducer and urea signals, we further investigated UilS contribution to the ability of *S. marcescens* to thrive in these conditions when forming part of a mixed bacterial population. The competition between the wild-type and *uilS* strains showed a significant disadvantage of the mutant when bacteria was exposed to urea. Moreover, this fitness difference was strengthened when both urea and AHL were present in the bacterial growth medium. A similar phenotype was determined when a co-culture of the wild-type and the *luxR* mutant stain was tested, although as expected, only urea but not AHL altered the fitness ratios obtained, reinforcing the regulatory role of AHL-dependent LuxR on *uilS* expression. Consistent with LuxR being the AHL-dependent transcriptional regulator of *luxI* and LuxI being dispensable for *uilS* transcriptional induction, the *luxI* strain exhibited fitness disadvantage compared to the wild-type strain when urea and AHL were present. In any case, we cannot rule out the potential participation of other LuxR-regulated genes which might also be playing a role in *S. marcescens* fitness.

The relationship between the ability to degrade AHL and bacterial fitness advantage can be explained by the adaptive superiority of a strain capable of modulating gene expression based on simultaneous detection of stress environmental signals and self-population density. This stands in contrast to bacteria where QS-dependent circuits are disrupted by action of QQ agents, resulting in a loss of this adaptive mechanism. In the interspecies interaction, we also demonstrated that when *S. marcescens* was confronted with *P. aeruginosa* as prey, *Serratia’s* killing capacity was abrogated by the deletion of either *uilS*, *luxR*, or *cpxR*. The contribution of LuxR- and CpxR-dependent UilS expression to the killing ability of *S. marcescens* was similar to the killing capacity conferred by the expression of the RcsB-dependent Type VI Secretion System when compared to the loss of killing capacity by either the *tssM* or the *rcsB* mutant strains used as control. To our knowledge, although we have not yet determined the fine mechanism that underlies the UilS-dependent killing phenotype, this is the first report that shows a bacterial interspecies killing mechanism that involves a QQ lactonase under the control of both QS and external stress cues.

In light of these results, we propose that CpxR- and LuxR-dependent control of UilS expression can introduce changes into a mixed population inhabited by *S. marcescens* by conferring competitive advantage during the development of a complex community. The ability of *S. marcescens* to customize adaptive responses based on the host environment, particularly in the presence of urea (a dominant component encountered by the pathogen during urinary tract infections), provides *S. marcescens* with an additional advantage for overcoming harsh conditions. Consistent with these observations, when testing *S. marcescens* in a mice model of CAUTI either by use of mono-inoculation or by a competitive assay, the *uilS* mutant strain was attenuated in its pathogenic capacity, showing a marked disadvantage to colonize the indwelling tubing as well as the host bladder. It has been recently shown that the composition of the urobiome can contribute to recurrence of urinary tract infection (UTI) and also influence bladder cancer development (44). The knowledge of the potential capacity of *Serratia* to modulate QS signals and alter the interactions of the urobiome can provide new tools to prevent and treat urinary tract diseases. In addition, and in contrast to antibiotics, inhibition of QS by QQ agents such as UilS is anticipated to selectively suppress virulence mechanisms in harmful bacteria without impeding their growth. Consequently, due to their low likelihood of promoting antibiotic-resistant bacterial strains, QS inhibitors are being considered as favorable candidates for combating bacterial infections.

**Fig 11:**
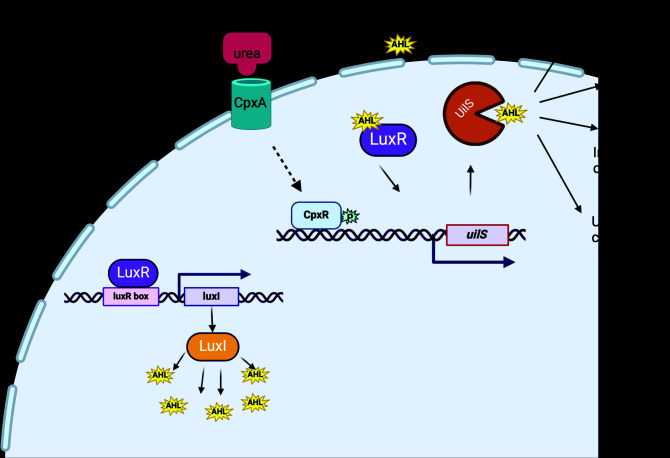
Proposed model for the AHL- and urea-dependent regulation of *S. marcescens* UilS expression and the resulting bacterial phenotypes. *uilS* is expressed at a basal transcriptional level, and this expression can be enhanced by an AHL-activated LuxR. When bacteria detect the presence of urea in the growth medium (probably by activation of CpxA, the CpxR-cognate stress sensor of the CpxAR two-component system), *uilS* transcription is further upregulated by the binding of an activated (phosphorylated) form of CpxR to the *uilS* promoter. UilS expression endows *S. marcescens* with the capacity of (self and non-self) AHL degradation, augments the bacterial fitness and killing capacity, and favors bladder colonization in a CAUTI mouse model of infection.

Interestingly, by phylogeny analysis, it becomes apparent that UilS homologs (with a deduced amino acid sequence identity in the 97%–100% range) are present in most *S. marcescens* clinical isolates, while the genomes derived from environmental sources lack a *uilS* gene. This strongly suggests that a host-associated powerful positive selective pressure has evolutionarily operated to maintain *uilS* in the genome of pathogenic *S. marcescens* strains. Moreover, although *luxR* is present in 100% of the analyzed strains, the presence or absence of *luxI* homologs does not appear to correlate with the presence of *luxR* or *luxI* homologs, or with the strain source. This reinforces the concept that *luxI* is not a necessary component in the regulatory cascade governing UilS expression. The proposed regulatory circuit and the UilS-associated phenotypes examined in this work are summarized in the scheme shown in Fig. 11.

In summary, our findings establish UilS as a key virulence factor in *S. marcescens*, highlighting its potential as a novel target for developing alternative antimicrobial strategies to combat infections caused by this pathogen.

## MATERIALS AND METHODS

### Bacterial strains and plasmids

The bacterial strains and plasmids used in this study are listed in Table S2 (see at http://ibr-conicet.gov.ar/wp-content/uploads/2024/09/mBio-Tuttobene-et-al-Supplemental-Material.pdf). Bacteria were routinely grown in Miller’s LB medium or on LB agar plates overnight at 30°C or 37°C. The antibiotics used were ampicillin (100 µg/mL), kanamycin (50 µg/mL), chloramphenicol (20 µg/mL), streptomycin (100 µg/mL), and gentamicin (15 µg/mL).

*S. marcescens* RM66262 has been classified as a urease-negative strain by the conventional Christensen urea agar test (45). The genome sequence search also revealed the lack of homologs to the urease complex cluster genes *ureABIEFGH* (17).

### RNA extraction, qRT-PCR, sequencing, and transcriptomic analysis

*S. marcescens* RM66262 was cultured in LB broth and incubated with agitation for 18 h at 37°C. Overnight cultures were then diluted 1:10 in fresh LB broth, LB broth supplemented with 0.2 M and 0.4 M urea, 1% LB 50% human urine or 0.1% 99.9% human urine, and incubated with agitation for 18 h at 37°C. A total of 250 µL of ice-cold 5% (vol/vol) water-saturated phenol (pH 5.5) in ethanol was added to 1 mL of the cultures to stop the degradation of RNA. Cells were centrifuged at 6,000 *g* for 5 min at 4°C and resuspended in 100 µL of 10 mM Tris-HCl and 1 mM EDTA (pH 8.0). The RNA extraction was performed using the Promega SV total RNA isolation kit, following the manufacturer’s instructions. Samples were confirmed to have no DNA contamination through PCR amplification of the 16S ribosomal DNA gene.

cDNA synthesis was performed using random hexamers, 2 mg of total RNA, and 1 U of SuperScript II reverse transcriptase (Invitrogen). Five microliter of a 1/10 dilution of each cDNA were used as the template for DNA amplification in qRT-PCR (20 µL), using primers for *uilS*, *prtA*, *fliA*, *fliC*, *flhD*, *slpE*, *slpD*, *lipB*, and *lipC* (Table S3, see at http://ibr-conicet.gov.ar/wp-content/uploads/2024/09/mBio-Tuttobene-et-al-Supplemental-Material.pdf). The reactions were carried out in the presence of HOT FIREPol EvaGreen qPCR Mix Plus (ROX; SOLIS BIODYNE) and monitored in real-time with the DLAB Accurate 96 real-time PCR system. The relative expression was calculated using the qBASE method (40). The qBASE method is a modification of the Ct method that considers multiple reference genes (*rpoD* and *gyrB*) and gene-specific amplification efficiencies. The average values were calculated from triplicate samples. Differences were determined by analysis of variance followed by Tukey’s multiple comparison test (*P* < 0.05) using GraphPad Prism (GraphPad software version 9.3.1, San Diego, CA, USA).

RNA sequencing was outsourced to Novegene (Novogene Corporation, Sacramento,CA, USA), where the RNA-seq library preparation (Illumina, San Diego, CA, USA) and HiSeq 2500 paired-end 150 bp sequencing were performed on three independent biological replicates in the of each condition. Trimming of low-quality bases at the ends of the reads to a minimum length of 100 bp and removing Illumina adaptor sequences was performed using Trimmomatic (46), yielding an average of 8.5 million paired reads per sample. FastQC was used to assess the quality of the reads before and after trimming. Burrows–Wheeler Alignment software was used to align the RNA-seq reads to the genome of *S. marcescens* RM66262 (47). The alignments were visualized using the Integrated Genome Viewer software (48). FeatureCounts (49) was used to calculate the read counts per gene, and differential expression analysis was performed using DEseq2 (50). Features exhibiting FDR < 0.05 and log2fold change >1 were considered statistically significant.

### Genetic manipulations

Insertion mutation in *uilS* (RT90_RS06020) was constructed with the pKNOCK-Cm suicide plasmid (51). An internal 500 bp region was amplified using primers uilS-F.BamHI and uilS-R.XhoI (see Table S3 at http://ibr-conicet.gov.ar/wp-content/uploads/2024/09/mBio-Tuttobene-et-al-Supplemental-Material.pdf). The purified PCR product was digested with the restriction enzymes indicated in the primer names and cloned into the pKNOCK-Cm plasmid. The resulting plasmids were introduced into competent *E. coli* SM10 λpir (52) cells by electroporation and then mobilized into *S. marcescens* RM66262 by conjugation. Insertional mutants were selected from chloramphenicol-resistant colonies, and chromosomal mutation was confirmed by PCR analysis using primers uilS-Fw and uilS-Rv.

*S. marcescens ΔluxI* and *ΔluxR* were constructed as follows. PCR was used to generate 500 bp of DNA upstream of either *luxI* (RT90_RS14900) or *luxR* (RT90_RS14905) using primers luxI-A and luxI-B and luxR-A and luxR-B, respectively (see Table S3 at http://ibr-conicet.gov.ar/wp-content/uploads/2024/09/mBio-Tuttobene-et-al-Supplemental-Material.zip), and ~500 bp of DNA downstream of *luxI* and *luxR* using primers luxI-C and luxI-D and luxR-C and luxR-D, respectively (see Table S4 at http://ibr-conicet.gov.ar/wp-content/uploads/2024/09/mBio-Tuttobene-et-al-Supplemental-Material.pdf). Through the splice by overlap extension (SOE)-PCR technique, both fragments served as primers for each other to generate a 1,000 bp product. The resulting DNA fragments were digested with the BamHI-SpeI restriction enzymes and ligated into the BamHI and SpeI sites of pKNG101 (53). pKNG101::*ΔluxI* and pKNG101::*ΔluxR* recombinant plasmids, contents, respectively, in the donor strain *E. coli* TOP10, were then mobilized into *S. marcescens* RM66262 by conjugation. Mutant strains were selected with streptomycin, and then high sucrose [15% (wt/vol)] allowed the isolation of mutants in which the deletion allele had replaced the wild-type copy. The deletion of *luxI* and *luxR* was confirmed by PCR using primers luxI-A and luxI-D, as well as luxR-Fw and luxR-Rv.

For complementation of the *S. marcescens uilS* mutant strain, the *uilS* gene was amplified by PCR from the *S. marcescens* wild-type strain chromosome using primers C.uilS-Fw (*Bam*HI site) and C.uilS-Rv (*Hind*III site; see Table S3 at http://ibr-conicet.gov.ar/wp-content/uploads/2024/09/mBio-Tuttobene-et-al-Supplemental-Material.pdf). The PCR product was cloned into the pSU36 plasmid (54) and introduced into the competent *E. coli* TOP10 strain. The construction was mobilized into *S. marcescens* RM66262 by electroporation.

For complementation of the *S. marcescens luxR* mutant strain, the *luxR* gene was amplified from the *S. marcescens* wild-type strain chromosome by PCR using primers C.luxR-Fw (*Xho*I site) and C.luxR-Rv (*Bam*HI site; see Table S3 at http://ibr-conicet.gov.ar/wp-content/uploads/2024/09/mBio-Tuttobene-et-al-Supplemental-Material.pdf). The PCR product was cloned into the pBB1-MCS1::lacI^q^ plasmid (55). Additionally, using primers C2.luxR-Fw (*Bam*HI site) and C2.luxR-Rv (*Hind*III site), the PCR product was cloned into the pSU36 plasmid (54). The construction pBB1-lacI^q^::*luxR* was mobilized into *S. marcescens* RM66262 strains by conjugation, while the construction pSU36::*luxR* was mobilized by electroporation. Two different complementation plasmids were used due to the incompatibility of the replication origins and resistance cassettes they possess.

### Transcriptional expression level analyses

To analyze the transcriptional levels of *uilS*, *luxI*, and *luxR*, the promoter regions of the genes were amplified by PCR using the primers P.uilS-Fw and P.uilS-Rv or P.uilS-Rv2, P.luxI-Fw and P.luxI-Rv, and P.luxR-Fw and P.luxR.Rv (see Table S3 at http://ibr-conicet.gov.ar/wp-content/uploads/2024/09/mBio-Tuttobene-et-al-Supplemental-Material.pdf). The purified PCR products were digested with the HindIII and XbaI (or SacI, when P.uilS-Rv2 was used) restriction enzymes and were ligated into the same sites of pPROBE(NT) (or of pPROBE(KT) to obtain p*PuilS*i-gfp) (56). The resulting plasmids were introduced into competent *E. coli* Top10 cells by transformation. The plasmids P*uilS*-gfp, P*uilS*i-gfp, P*luxI*-gfp, and P*luxR*-gfp were mobilized by conjugation into the *S. marcescens* strains.

### *uilS*, *luxI*, and *luxR* gene expression assays

Cultures of *S. marcescens* strains carrying the pP*uilS*-gfp, pP*uilS*i-gfp, pP*luxI*-gfp, or pP*luxR*-gfp reporter plasmids were grown with shaking overnight at 37°C. The bacterial cultures were washed with 1× phosphate buffered saline (PBS). Next, 1/100 dilutions were made in LB supplemented with kanamycin, and 100 µL volumes of the mixtures were incubated in a 96-microwell plate at 37°C with agitation for 16 h. Optical density at 600 nm (OD600) and GFP fluorescence [excitation wavelength (λexcitation) of 485 nm and emission wavelength (λemission) at 528 nm] were determined using a 96-microwell plate reader (Synergy 2). Transcriptional activity was calculated as the ratio of GFP fluorescence and OD600 (FU/OD600). The means and SDs for three independent assays performed in duplicate in each case were calculated.

### Growth of *S. marcescens* on different supplemented LB broth

To test the ability of the *S. marcescens* wild type, *uilS*, and *uilS* pSU36::*uilS* to grow at 37°C in agitation, 1/100 dilutions of overnight cultures grown in LB were inoculated in LB, LB supplemented with 0.2 or 0.4 M urea, 1% LB 50% human urine, 0.1% LB 99.9% human urine, and grown at 37°C and 200 rpm. For 18 h, OD600 nm was determined, determining it every hour.

### AHLs detection using biosensor strains

*A. tumefaciens* NT1 (pZLR4) was used to detect the presence of AHLs in filtered supernatants of *S. marcescens* strains. The *A. tumefaciens* NT1 (pZLR4) AHL biosensor, which contains a plasmid-localized *traG-lacZ* fusion (pZLR4) (57, 58), responds to AHLs of chain lengths ranging from C6 to C12. Ten microliter of filtered supernatants was loaded in a central well of LB 0.7% agar plates previously inoculated with 250 µL of the *A. tumefaciens* culture and 30 µL of 20 mg/mL X-gal per 20 mL melted agar. The plates were then incubated at 30°C for 24 h. *C. violaceum* VIR07 (59) was also used to detect the presence of AHLs in *S. marcescens* strains. Violacein is inducible by compounds with N-acyl side chains from C10 to C16 (59).

### Determination of secreted Hcp protein levels by immunodetection

The determination of protein levels by immunodetection was performed as described (60). Briefly, 10 mL of cultures was grown overnight with good aeration in LB at 37°C and normalized by OD600. Cultures were centrifuged for 5 min at 5,000 *g*, and the supernatant was separated. The supernatant was filtered with 0.2 µm acetate-cellulose filters, precipitated with 12% trichloroacetic acid for 2 h at 4°C, and centrifuged for 30 min at 30,000 *g*. The precipitated secreted proteins were resuspended in the protein sample buffer. Twenty microgram of total protein was loaded onto 18% SDS-PAGE gels and transferred to Hybond-ECL nitrocellulose membranes. The membranes were blocked for 1 h with 5% non-fat milk and 0.1% Tris-Buffer Saline (TBS) and washed twice in TBS for 10 min. Then, the blots were incubated with *S. marcescens* anti-Hcp rabbit polyclonal antibodies, washed twice in TBS, and finally incubated with anti-rabbit secondary antibody conjugated to the enzyme alkaline phosphatase (Sigma) at a 1/3,500 dilution in TBS. This was followed by three washes of 10 min each with TBS. For development, the membrane was incubated with 3 mL of the 100 mM Tris pH 9.0, 100 mM NaCl, and 25 mM MgCl_2_ solution containing 0.15 mg/mL 5-bromo-4-chloro-3-indolyl phosphate (BCIP) and 0.3 mg/mL nitro blue tetrazolium (NBT) dissolved in 70% (vol/vol) N,N-dimethylformamide, until color development. The assay was repeated three times.

### Expression and purification of CpxR-6His

Purification of CpxR-6His was done by recombinant expression of the tagged protein followed by affinity purification protocols as described in Bruna et al. (21).

### Protein-DNA interaction analysis

To evaluate the interaction of CpxR with the promoter region of the *uilS* gene, EMSA was performed as described by Bruna et al. (21). We used a probe containing 500 pb upstream of ATG site of *uils* gene and 0.8 µM of purified CpxR-6xHis. The non-specific DNA was assayed using a 441 bp PCR probe corresponding to the *nucA* gene from *S. marcescens*. The primers used to amplify the P*uils* region and *nucA* are listed in Table S4 (see at http://ibr-conicet.gov.ar/wp-content/uploads/2024/09/mBio-Tuttobene-et-al-Supplemental-Material.pdf). After electrophoresis, the gels were stained with SYBR green (Invitrogen). DNA and protein–DNA complexes were detected and captured using a Typhoon FLA7000 laser scanner (GE Healthcare).

### Fitness assay

Bacterial cells grown overnight were normalized to an optical density at 600 nm (OD600) of 0.5 and mixed at a 1:1 ratio. Twenty-five microliter of this mixture was spotted onto a prewarmed LB, LB 0.4 M urea, LB 10 µM C10-AHL, or LB 0.4 M urea 10 µM C10-AHL agar plate and incubated at 37°C for 6 or 24 h, as indicated. Cells were recovered from the spot and resuspended in 1 mL LB broth. Serial dilutions were plated out on antibiotic selection medium. The recovery of viable cells is reported as the total number recovered per coculture spot. The results for each experiment are the average values of an assay performed in triplicate and independently repeated at least four times.

### Antibacterial killing assay

Killing assays were performed as described previously (39), with modifications as follows. Bacterial cells grown overnight were normalized to an optical density at 600 nm (OD600) of 0.5 and mixed at a 5:1 or 10:1 (attacker/target) ratio as indicated in each figure legend. Twenty-five microliter of this mixture was spotted onto a prewarmed agar plate and incubated at 37°C for 6 h, as indicated. Cells were recovered from the spot and resuspended in 1 mL LB broth. Serial dilutions were plated out on antibiotic selection medium, using kanamycin for *P. aeruginosa* strain PAO1. Controls consisted of PAO1 strain in LB. The recovery of viable cells is reported as the total number recovered per coculture spot. The results for each experiment are the average values of an assay performed in triplicate and independently repeated at least four times.

### Murine model of *S. marcescens* CAUTI

Six- to 8-week-old female C57BL/6 mice were obtained from Charles River Laboratories. Mice were transurethrally implanted with a small piece of silicone tubing (catheter implant, 4 to 5 mm piece of RenaSil Silicone Rubber Tubing. SIL 025, 0.025 inches outer diameter × 0.012 inches inner diameter) and inoculated as described (61, 62). Briefly, mice were anesthetized by inhalation of 4% isoflurane, and a 4 to 5 mm piece of silicone tubing (catheter) was placed in the bladder via transurethral insertion. *S. marcescens* strains were prepared for inoculation as follows: static growth at 37°C 18 h followed by centrifugation at 6,500 rpm for 5 min, washing twice in 1× PBS, and resuspension in PBS to the final inoculum. When indicated, mice were infected immediately following implant placement with ∼1 × 10^8^ CFUs bacteria in 50 µL via transurethral inoculation. At 24 h post-infection, mice were euthanized, and kidneys, bladders, and implants were aseptically removed. Implants were placed in 1 mL PBS, and bacteria were detached in an ultrasonic benchtop water bath. The bacterial load present in each tissue was determined by homogenizing each organ in PBS and plating serial dilutions on LB agar supplemented with antibiotics when appropriate. For competition studies, overnight cultures of wild-type and mutant strains were mixed ∼1:1 and enumerated by serial dilution and agar plating in Kan 50 µg/mL or Cm 20 µg/mL plates to determine input numbers. Bacterial enumeration from infected animals was similarly done to determine output ratios to calculate the competitive index. The CI was defined as the mutant-to-wild-type ratio (CFU mutant/CFU wild type) within the output sample, divided by the corresponding ratio in the inoculum. Statistical significance was evaluated using the One-sample Wilcoxon test using Graph Pad Prism. All CAUTI studies were performed in accordance with the guidelines of the Washington University School of Medicine Institutional Animal Care and Use Committee, and we have complied with all relevant ethical regulations. Mice were housed with a cycle consisting of 12 h of light and dark with access to standard food and water *ad libitum*.

### Phylogenetic analysis

The 21 complete genome sequences of *S. marcescens* were retrieved from the NCBI Genome website (https:// www.ncbi.nlm.nih.gov/genome) as of 11 October 2023. The phylogenetic tree based on the whole-genome alignments was constructed using REALPHY 1.13 (63). The classification of *luxI* and gene arrangement around the class I *luxR* homolog were performed according to the method published by Sakuraoka et al. (64). The search of the amino acid identity percentage in relation to *S. marcescens* RM66262 was carried through BlastP analysis (https://blast.ncbi.nlm.nih.gov/Blast.cgi?PAGE=Proteins).

## References

[1] Hense BA, Schuster M. 2015. Core principles of bacterial autoinducer systems. Microbiol Mol Biol Rev 79:153–169. doi:10.1128/MMBR.00024-14 25694124 PMC4402962

[2] Mukherjee S, Bassler BL. 2019. Bacterial quorum sensing in complex and dynamically changing environments. Nat Rev Microbiol 17:371–382. doi:10.1038/s41579-019-0186-5 30944413 PMC6615036

[3] Papenfort K, Bassler BL. 2016. Quorum sensing signal–response systems in Gram-negative bacteria. Nat Rev Microbiol 14:576–588. doi:10.1038/nrmicro.2016.89 27510864 PMC5056591

[4] Fuqua C, Winans SC. 1996. Conserved cis-acting promoter elements are required for density-dependent transcription of Agrobacterium tumefaciens conjugal transfer genes. J Bacteriol 178:435–440. doi:10.1128/jb.178.2.435-440.1996 8550463 PMC177675

[5] Sikdar R, Elias M. 2020. Quorum quenching enzymes and their effects on virulence, biofilm, and microbiomes: a review of recent advances. Expert Rev Anti Infect Ther 18:1221–1233. doi:10.1080/14787210.2020.1794815 32749905 PMC7705441

[6] Grandclément C, Tannières M, Moréra S, Dessaux Y, Faure D. 2016. Quorum quenching: role in nature and applied developments. FEMS Microbiol Rev 40:86–116. doi:10.1093/femsre/fuv038 26432822

[7] Khanna A, Khanna M, Aggarwal A. 2013. Serratia marcescens- a rare opportunistic nosocomial pathogen and measures to limit its spread in hospitalized patients. J Clin Diagn Res 7:243–246. doi:10.7860/JCDR/2013/5010.2737 23543704 PMC3592283

[8] Tavares-Carreon F, De Anda-Mora K, Rojas-Barrera IC, Andrade A. 2023. Serratia marcescens antibiotic resistance mechanisms of an opportunistic pathogen: a literature review. PeerJ 11:e14399. doi:10.7717/peerj.14399 36627920 PMC9826615

[9] Hines DA, Saurugger PN, Ihler GM, Benedik MJ. 1988. Genetic analysis of extracellular proteins of Serratia marcescens. J Bacteriol 170:4141–4146. doi:10.1128/jb.170.9.4141-4146.1988 2842305 PMC211420

[10] Eberl L, Winson MK, Sternberg C, Stewart GS, Christiansen G, Chhabra SR, Bycroft B, Williams P, Molin S, Givskov M. 1996. Involvement of N-acyl-l-homoserine lactone autoinducers in controlling the multicellular behaviour of Serratia liquefaciens. Mol Microbiol 20:127–136. doi:10.1111/j.1365-2958.1996.tb02495.x 8861211

[11] Horng Y-T, Deng S-C, Daykin M, Soo P-C, Wei J-R, Luh K-T, Ho S-W, Swift S, Lai H-C, Williams P. 2002. The LuxR family protein SpnR functions as a negative regulator of N-acylhomoserine lactone-dependent quorum sensing in Serratia marcescens. Mol Microbiol 45:1655–1671. doi:10.1046/j.1365-2958.2002.03117.x 12354232

[12] Rice SA, Koh KS, Queck SY, Labbate M, Lam KW, Kjelleberg S. 2005. Biofilm formation and sloughing in Serratia marcescens are controlled by quorum sensing and nutrient cues. J Bacteriol 187:3477–3485. doi:10.1128/JB.187.10.3477-3485.2005 15866935 PMC1111991

[13] Van Houdt R, Givskov M, Michiels CW. 2007. Quorum sensing in Serratia. FEMS Microbiol Rev 31:407–424. doi:10.1111/j.1574-6976.2007.00071.x 17459113

[14] European Centre for Disease Prevention and Control. 2024. Healthcare-associated infections acquired in intensive care units

[15] Barchitta M, Maugeri A, Favara G, Lio RMS, La Rosa MC, D’Ancona F, Agodi A, SPIN-UTI network. 2023. The intertwining of healthcare-associated infections and COVID-19 in Italian intensive care units: an analysis of the SPIN-UTI project from 2006 to 2021. J Hosp Infect 140:124–131. doi:10.1016/j.jhin.2023.07.021 37562591

[16] Sarigul N, Korkmaz F, Kurultak İ. 2019. A new artificial urine protocol to better imitate human urine. Sci Rep 9:20159. doi:10.1038/s41598-019-56693-4 31882896 PMC6934465

[17] Bruna RE, Revale S, García Véscovi E, Mariscotti JF. 2015. Draft whole-genome sequence of Serratia marcescens strain RM66262, isolated from a patient with a urinary tract infection. Genome Announc 3:e01423-15. doi:10.1128/genomeA.01423-15 PMC4669405 26634764

[18] Tuttobene MR, Schachter J, Álvarez CL, Saffioti NA, Leal Denis MF, Kessler H, García Véscovi E, Schwarzbaum PJ. 2023. ShlA toxin of Serratia induces P2Y2- and α5β1-dependent autophagy and bacterial clearance from host cells. J Biol Chem 299:105119. doi:10.1016/j.jbc.2023.105119 37527778 PMC10474472

[19] Aziz RK, Bartels D, Best AA, DeJongh M, Disz T, Edwards RA, Formsma K, Gerdes S, Glass EM, Kubal M, et al.. 2008. The RAST server: rapid annotations using subsystems technology. BMC Genomics 9:75. doi:10.1186/1471-2164-9-75 18261238 PMC2265698

[20] Nakahama K, Yoshimura K, Marumoto R, Kikuchi M, Lee IS, Hase T, Matsubara H. 1986. Cloning and sequencing of Serratia protease gene. Nucleic Acids Res 14:5843–5855. doi:10.1093/nar/14.14.5843 3016665 PMC311595

[21] Bruna RE, Molino MV, Lazzaro M, Mariscotti JF, García Véscovi E. 2018. CpxR-dependent thermoregulation of Serratia marcescens PrtA metalloprotease expression and its contribution to bacterial biofilm formation. J Bacteriol 200:1–18. doi:10.1128/JB.00006-18 PMC5869481 29378892

[22] Stella NA, Callaghan JD, Zhang L, Brothers KM, Kowalski RP, Huang JJ, Thibodeau PH, Shanks RMQ. 2017. SlpE is a calcium-dependent cytotoxic metalloprotease associated with clinical isolates of Serratia marcescens. Res Microbiol 168:567–574. doi:10.1016/j.resmic.2017.03.006 28366837 PMC5503780

[23] Shanks RMQ, Stella NA, Hunt KM, Brothers KM, Zhang L, Thibodeau PH. 2015. Identification of SlpB, a cytotoxic protease from Serratia marcescens. Infect Immun 83:2907–2916. doi:10.1128/IAI.03096-14 25939509 PMC4468537

[24] Castelli ME, Fedrigo GV, Clementín AL, Ielmini MV, Feldman MF, Véscovi EG. 2008. Enterobacterial common antigen integrity is a checkpoint for flagellar biogenesis in Serratia marcescens. J Bacteriol 190:213–220. doi:10.1128/JB.01348-07 17981971 PMC2223741

[25] Akatsuka H, Binet R, Kawai E, Wandersman C, Omori K. 1997. Lipase secretion by bacterial hybrid ATP-binding cassette exporters: molecular recognition of the LipBCD, PrtDEF, and HasDEF exporters. J Bacteriol 179:4754–4760. doi:10.1128/jb.179.15.4754-4760.1997 9244262 PMC179321

[26] Akatsuka H, Kawai E, Omori K, Shibatani T. 1995. The three genes lipB, lipC, and lipD involved in the extracellular secretion of the Serratia marcescens lipase which lacks an N-terminal signal peptide. J Bacteriol 177:6381–6389. doi:10.1128/jb.177.22.6381-6389.1995 7592412 PMC177487

[27] López M, Mayer C, Fernández-García L, Blasco L, Muras A, Ruiz FM, Bou G, Otero A, Tomás M, GEIH-GEMARA (SEIMC). 2017. Quorum sensing network in clinical strains of A. baumannii: AidA is a new quorum quenching enzyme. PLoS One 12:e0174454. doi:10.1371/journal.pone.0174454 28328989 PMC5362224

[28] Cha C, Gao P, Chen YC, Shaw PD, Farrand SK. 1998. Production of acyl-homoserine lactone quorum-sensing signals by gram-negative plant-associated bacteria. Mol Plant Microbe Interact 11:1119–1129. doi:10.1094/MPMI.1998.11.11.1119 9805399

[29] Morohoshi T, Shiono T, Takidouchi K, Kato M, Kato N, Kato J, Ikeda T. 2007. Inhibition of quorum sensing in Serratia marcescens AS-1 by synthetic analogs of N-acylhomoserine lactone. Appl Environ Microbiol 73:6339–6344. doi:10.1128/AEM.00593-07 17675425 PMC2075062

[30] Nain Z, Adhikari UK, Abdulla F, Hossain N, Barman NC, Mansur FJ, Azakami H, Karim MM. 2020. Computational prediction of active sites and ligands in different AHL quorum quenching lactonases and acylases. J Biosci 45:1–19. doi:10.1007/s12038-020-0005-1 32020908

[31] Shang Z, Wang H, Zhou S, Chu W. 2014. Characterization of N-Acyl-homoserine lactones (AHLs)-deficient clinical isolates of Pseudomonas aeruginosa. Indian J Microbiol 54:158–162. doi:10.1007/s12088-014-0449-9 25320416 PMC4188499

[32] Begic S, Worobec EA. 2006. Regulation of Serratia marcescens ompF and ompC porin genes in response to osmotic stress, salicylate, temperature and pH. Microbiol (Reading) 152:485–491. doi:10.1099/mic.0.28428-0 16436436

[33] Lin Z, Cai X, Chen M, Ye L, Wu Y, Wang X, Lv Z, Shang Y, Qu D. 2017. Virulence and stress responses of Shigella flexneri regulated by PhoP/PhoQ. Front Microbiol 8:2689. doi:10.3389/fmicb.2017.02689 29379483 PMC5775216

[34] Romanowski EG, Stella NA, Romanowski JE, Yates KA, Dhaliwal DK, St Leger AJ, Shanks RMQ. 2021. The Rcs stress response system regulator GumB modulates Serratia marcescens-induced inflammation and bacterial proliferation in a rabbit keratitis model and cytotoxicity in vitro. Infect Immun 89:e0011121. doi:10.1128/IAI.00111-21 33820815 PMC8281226

[35] Castelli ME, Véscovi EG. 2011. The Rcs signal transduction pathway is triggered by enterobacterial common antigen structure alterations in Serratia marcescens. J Bacteriol 193:63–74. doi:10.1128/JB.00839-10 20971912 PMC3019949

[36] Horng YT, Chang KC, Liu YN, Lai HC, Soo PC. 2010. The RssB/RssA two-component system regulates biosynthesis of the tripyrrole antibiotic, prodigiosin, in Serratia marcescens. Int J Med Microbiol 300:304–312. doi:10.1016/j.ijmm.2010.01.003 20347390

[37] Liu J, Obi IR, Thanikkal EJ, Kieselbach T, Francis MS. 2011. Phosphorylated CpxR restricts production of the RovA global regulator in Yersinia pseudotuberculosis. PLoS One 6:e23314. doi:10.1371/journal.pone.0023314 21876746 PMC3158067

[38] Reglinski M, Monlezun L, Coulthurst SJ. 2023. The accessory protein TagV is required for full Type VI secretion system activity in Serratia marcescens. Mol Microbiol 119:326–339. doi:10.1111/mmi.15027 36627840 PMC7614798

[39] Lazzaro M, Feldman MF, García Véscovi E. 2017. A transcriptional regulatory mechanism finely tunes the firing of type VI secretion system in response to bacterial enemies. mBio 8:1–17. doi:10.1128/mBio.00559-17 PMC5565961 28830939

[40] Basler M. 2015. Type VI secretion system: secretion by a contractile nanomachine. Philos Trans R Soc Lond B Biol Sci 370:20150021. doi:10.1098/rstb.2015.0021 26370934 PMC4632598

[41] Felisberto-Rodrigues C, Durand E, Aschtgen M-S, Blangy S, Ortiz-Lombardia M, Douzi B, Cambillau C, Cascales E. 2011. Towards a structural comprehension of bacterial type VI secretion systems: characterization of the TssJ-TssM complex of an Escherichia coli pathovar. PLoS Pathog 7:e1002386. doi:10.1371/journal.ppat.1002386 22102820 PMC3213119

[42] Vogt SL, Raivio TL. 2012. Just scratching the surface: an expanding view of the Cpx envelope stress response. FEMS Microbiol Lett 326:2–11. doi:10.1111/j.1574-6968.2011.02406.x 22092948

[43] Conover MS, Flores-Mireles AL, Hibbing ME, Dodson K, Hultgren SJ. 2015. Establishment and characterization of UTI and CAUTI in a mouse model. J Vis Exp:e52892. doi:10.3791/52892 26132341 PMC4544389

[44] Bučević Popović V, Šitum M, Chow CET, Chan LS, Roje B, Terzić J. 2018. The urinary microbiome associated with bladder cancer. Sci Rep 8:12157. doi:10.1038/s41598-018-29054-w 30108246 PMC6092344

[45] Biochemical tests for the identification of aerobic bacteria, p 3. 2016. In Clinical microbiology procedures handbook. ASM Press.

[46] Bolger AM, Lohse M, Usadel B. 2014. Trimmomatic: a flexible trimmer for Illumina sequence data. Bioinformatics 30:2114–2120. doi:10.1093/bioinformatics/btu170 24695404 PMC4103590

[47] Li H, Durbin R. 2010. Fast and accurate long-read alignment with Burrows–Wheeler transform. Bioinformatics 26:589–595. doi:10.1093/bioinformatics/btp698 20080505 PMC2828108

[48] Robinson JT, Thorvaldsdóttir H, Winckler W, Guttman M, Lander ES, Getz G, Mesirov JP. 2011. Integrative genomics viewer. Nat Biotechnol 29:24–26. doi:10.1038/nbt.1754 21221095 PMC3346182

[49] Liao Y, Smyth GK, Shi W. 2014. featureCounts: an efficient general purpose program for assigning sequence reads to genomic features. Bioinformatics 30:923–930. doi:10.1093/bioinformatics/btt656 24227677

[50] Love MI, Huber W, Anders S. 2014. Moderated estimation of fold change and dispersion for RNA-seq data with DESeq2. Genome Biol 15:550. doi:10.1186/s13059-014-0550-8 25516281 PMC4302049

[51] Alexeyev MF. 1999. The pKNOCK series of broad-host-range mobilizable suicide vectors for gene knockout and targeted DNA insertion into the chromosome of gram-negative bacteria. BioTechniques 26:824–826. doi:10.2144/99265bm05 10337469

[52] Simon R, Priefer U, Pühler A. 1983. A broad host range mobilization system for in vivo genetic engineering: transposon mutagenesis in gram negative bacteria. Nat Biotechnol 1:784–791. doi:10.1038/nbt1183-784

[53] Kaniga K, Delor I, Cornelis GR. 1991. A wide-host-range suicide vector for improving reverse genetics in Gram-negative bacteria: inactivation of the blaA gene of Yersinia enterocolitica. Gene 109:137–141. doi:10.1016/0378-1119(91)90599-7 1756974

[54] Bartolomé B, Jubete Y, Martínez E, de la Cruz F. 1991. Construction and properties of a family of pACYC184-derived cloning vectors compatible with pBR322 and its derivatives. Gene 102:75–78. doi:10.1016/0378-1119(91)90541-i 1840539

[55] Barchiesi J, Castelli ME, Di Venanzio G, Colombo MI, García Véscovi E. 2012. The Phop/PhoQ system and Its role in Serratia marcescens pathogenesis. J Bacteriol 194:2949–2961. doi:10.1128/JB.06820-11 22467788 PMC3370626

[56] Miller WG, Leveau JHJ, Lindow SE. 2000. Improved gfp and inaZ broad-host-range promoter-probe vectors. Mol Plant Microbe Interact 13:1243–1250. doi:10.1094/MPMI.2000.13.11.1243 11059491

[57] Farrand SK, Qin Y, Oger P. 2022. Quorum-sensing system of Agrobacterium plasmids: analysis and utility. Meth Enzymol:452–484. doi:10.1016/S0076-6879(02)58108-8 12474406

[58] Steindler L, Venturi V. 2007. Detection of quorum-sensing N-acyl homoserine lactone signal molecules by bacterial biosensors. FEMS Microbiol Lett 266:1–9. doi:10.1111/j.1574-6968.2006.00501.x 17233715

[59] Morohoshi T, Kato M, Fukamachi K, Kato N, Ikeda T. 2008. N-Acylhomoserine lactone regulates violacein production in Chromobacterium violaceum type strain ATCC 12472. FEMS Microbiol Lett 279:124–130. doi:10.1111/j.1574-6968.2007.01016.x 18177311

[60] Di Venanzio G, Stepanenko TM, García Véscovi E. 2014. Serratia marcescens ShlA pore-forming toxin is responsible for early induction of autophagy in host cells and is transcriptionally regulated by RcsB. Infect Immun 82:3542–3554. doi:10.1128/IAI.01682-14 24914224 PMC4187834

[61] Guiton PS, Hung CS, Hancock LE, Caparon MG, Hultgren SJ. 2010. Enterococcal biofilm formation and virulence in an optimized murine model of foreign body-associated urinary tract infections. Infect Immun 78:4166–4175. doi:10.1128/IAI.00711-10 20696830 PMC2950371

[62] Di Venanzio G, Flores-Mireles AL, Calix JJ, Haurat MF, Scott NE, Palmer LD, Potter RF, Hibbing ME, Friedman L, Wang B, Dantas G, Skaar EP, Hultgren SJ, Feldman MF. 2019. Urinary tract colonization is enhanced by a plasmid that regulates uropathogenic Acinetobacter baumannii chromosomal genes. Nat Commun 10:2763. doi:10.1038/s41467-019-10706-y 31235751 PMC6591400

[63] Bertels F, Silander OK, Pachkov M, Rainey PB, van Nimwegen E. 2014. Automated reconstruction of whole-genome phylogenies from short-sequence reads. Mol Biol Evol 31:1077–1088. doi:10.1093/molbev/msu088 24600054 PMC3995342

[64] Sakuraoka R, Suzuki T, Morohoshi T. 2019. Distribution and genetic diversity of genes involved in quorum sensing and prodigiosin biosynthesis in the complete genome sequences of serratia marcescens. Genome Biol Evol 11:931–936. doi:10.1093/gbe/evz046 30840067 PMC6433178

